# Identification of Potential Modulators of a Pathogenic G Protein-Gated Inwardly Rectifying K^+^ Channel 4 Mutant: *In Silico* Investigation in the Context of Drug Discovery for Hypertension

**DOI:** 10.3390/molecules28247946

**Published:** 2023-12-05

**Authors:** Eleni Pitsillou, Alexander N. O. Logothetis, Julia J. Liang, Assam El-Osta, Andrew Hung, Asmaa S. AbuMaziad, Tom C. Karagiannis

**Affiliations:** 1 Epigenomic Medicine Laboratory at prospED Polytechnic, Carlton, VIC 3053, Australia; 2 School of Science, STEM College, RMIT University, Melbourne, VIC 3001, Australia; 3 Department of Microbiology and Immunology, The University of Melbourne, Parkville, VIC 3010, Australia; 4 Epigenetics in Human Health and Disease Program, Baker Heart and Diabetes Institute, 75 Commercial Road, Prahran, VIC 3004, Australia; 5 Department of Diabetes, Central Clinical School, Monash University, Melbourne, VIC 3004, Australia; 6 Department of Medicine and Therapeutics, The Chinese University of Hong Kong, Sha Tin, Hong Kong SAR, China; 7 Hong Kong Institute of Diabetes and Obesity, Prince of Wales Hospital, The Chinese University of Hong Kong, 3/F Lui Che Woo Clinical Sciences Building, 30-32 Ngan Shing Street, Sha Tin, Hong Kong SAR, China; 8 Li Ka Shing Institute of Health Sciences, The Chinese University of Hong Kong, Sha Tin, Hong Kong SAR, China; 9 Biomedical Laboratory Science, Department of Technology, Faculty of Health, University College Copenhagen, 1799 Copenhagen, Denmark; 10 Department of Pediatrics, College of Medicine Tucson, The University of Arizona, Tucson, AZ 85724, USA; 11 Department of Clinical Pathology, The University of Melbourne, Parkville, VIC 3010, Australia

**Keywords:** hypertension, primary aldosteronism, inwardly rectifying K^+^ channels, GIRK4, pathogenic mutations

## Abstract

Genetic abnormalities have been associated with primary aldosteronism, a major cause of secondary hypertension. This includes mutations in the *KCNJ5* gene, which encodes G protein-gated inwardly rectifying K^+^ channel 4 (GIRK4). For example, the substitution of glycine with glutamic acid gives rise to the pathogenic GIRK4^G151E^ mutation, which alters channel selectivity, making it more permeable to Na^+^ and Ca^2+^. While tertiapin and tertiapin-Q are well-known peptide inhibitors of the GIRK4^WT^ channel, clinically, there is a need for the development of selective modulators of mutated channels, including GIRK4^G151E^. Using *in silico* methods, including homology modeling, protein–peptide docking, ligand-binding site prediction, and molecular docking, we aimed to explore potential modulators of GIRK4^WT^ and GIRK4^G151E^. Firstly, protein–peptide docking was performed to characterize the binding site of tertiapin and its derivative to the GIRK4 channels. In accordance with previous studies, the peptide inhibitors preferentially bind to the GIRK4^WT^ channel selectivity filter compared to GIRK4^G151E^. A ligand-binding site analysis was subsequently performed, resulting in the identification of two potential regions of interest: the central cavity and G-loop gate. Utilizing curated chemical libraries, we screened over 700 small molecules against the central cavity of the GIRK4 channels. Flavonoids, including luteolin-7-O-rutinoside and rutin, and the macrolides rapamycin and troleandomycin bound strongly to the GIRK4 channels. Similarly, xanthophylls, particularly luteoxanthin, bound to the central cavity with a strong preference towards the mutated GIRK4^G151E^ channel compared to GIRK4^WT^. Overall, our findings suggest potential lead compounds for further investigation, particularly luteoxanthin, that may selectively modulate GIRK4 channels.

## 1. Introduction

Primary aldosteronism, which is characterized by the autonomous overproduction of aldosterone, is the most common cause of secondary hypertension [1,2]. The main causes of primary aldosteronism include aldosterone-producing adenomas (APA) and bilateral adrenal hyperplasia (BAH) [1,3,4]. The pathogenesis of primary aldosteronism predominantly involves somatic mutations that arise in genes expressed in zona glomerulosa cells, inducing the inappropriate production of aldosterone [5,6]. Sporadic cases of primarily aldosteronism are largely due to somatic mutations, while familial hyperaldosteronism (FH) results from inherited germline mutations [4]. Familial hyperaldosteronism is an autosomal-dominant disorder with various subtypes [4].

Pathogenic missense mutations in the *KCNJ5* gene have been detected in patients with primary aldosteronism, particularly FH-III [7,8]. The *KCNJ5* gene encodes the G protein-activated inward rectifier potassium (K^+^) channel 4 (GIRK4 or Kir3.4) [8]. G protein-coupled inward rectifier K^+^ (GIRK) channels belong to the larger inward rectifier K^+^ (Kir) channel family, and four subunits have been identified: GIRK1-4 [9]. Under physiological conditions, GIRK channels play an important role in regulating cell excitability and the resting membrane potential [10]. The activation of GIRK channels occurs when certain ligands, such as neurotransmitters and hormones, bind to their cognate G protein-coupled receptors [11]. This results in the dissociation of the βγ subunits of pertussis toxin-sensitive G proteins, which bind to and activate the GIRK channel [11,12]. Once the channel is opened, the outward flow of K^+^ leads to hyperpolarization of the membrane and a reduction in cell excitability [13].

The GIRK4 channel has been found to exist as a homotetrameric complex in the adrenal cortex, which produces aldosterone [14]. Most of the mutations in GIRK4 have been reported to occur in or within close proximity to the K^+^ selectivity filter, producing nonselective cation channels [15]. The influx of cations, including Na^+^, results in membrane depolarization and the activation of voltage-gated Ca^2+^ channels [15]. The increase in cytosolic Ca^2+^ promotes aldosterone secretion and the formation of adenomas [15].

The pathogenicity of the GIRK4^G151E^ mutation has previously been reported in individuals diagnosed with early onset hypertension and primary aldosteronism [7,16]. In a study by Scholl et al., the GIRK4^G151E^ mutant channel was found to have significantly higher Na^+^ conductance compared to the GIRK4^G151R^ variant [7]. It was suggested that the increased Na^+^-dependent cell lethality limits the adrenocortical cell mass, accounting for the distinct phenotype observed among patients [7].

The current treatment options for primary aldosteronism include mineralocorticoid receptor antagonists, such as spironolactone or eplerenone, as well as adrenalectomy [1,3,4]. Molecules, such as ethanol and naringin, have been found to activate GIRK channels in a G-protein-independent manner [17,18]. Kaufmann et al. developed and characterized ML297, the first potent and selective GIRK activator [19]. Structure–activity relationship (SAR) studies have been performed to identify novel selective modulators of GIRK channels using ML297 as a chemical scaffold [20,21]. Furthermore, the inhibitory activity of Ba^2+^, tertiapin, various antidepressant drugs, and the D1 dopamine receptor antagonist SCH23390 against GIRK channels has been reported [10,22,23]. Nonetheless, there is a clinical need for the development of direct inhibitors of mutant GIRK4 channels that can be used as diagnostic and therapeutic tools [4]. Several studies have reported the sensitivity of mutant GIRK4 channels to inhibitors of Na^+^ and Ca^2+^ transporting proteins and macrolide antibiotics; however, their mechanisms of action require further investigation [4].

In this study, *in silico* methods were used to explore the binding characteristics of peptide inhibitors and small molecules to homotetrameric GIRK4^WT^ and GIRK4^G151E^ channels. Homology models were constructed using the human *KCNJ5* amino acid sequence and cryo-EM structure of the mouse GIRK2 channel as a template. Potential binding sites, including the selectivity filter, central cavity, and G-loop gate regions, were identified and formed the basis of the molecular docking analyses. Using a selection of compounds with reported inhibitory activity against mutated GIRK4 channels and curated chemical libraries, molecular docking was performed to identify potential modulators of the GIRK4^WT^ and GIRK4^G151E^ channels.

## 2. Results and Discussion

**Figure 1:**
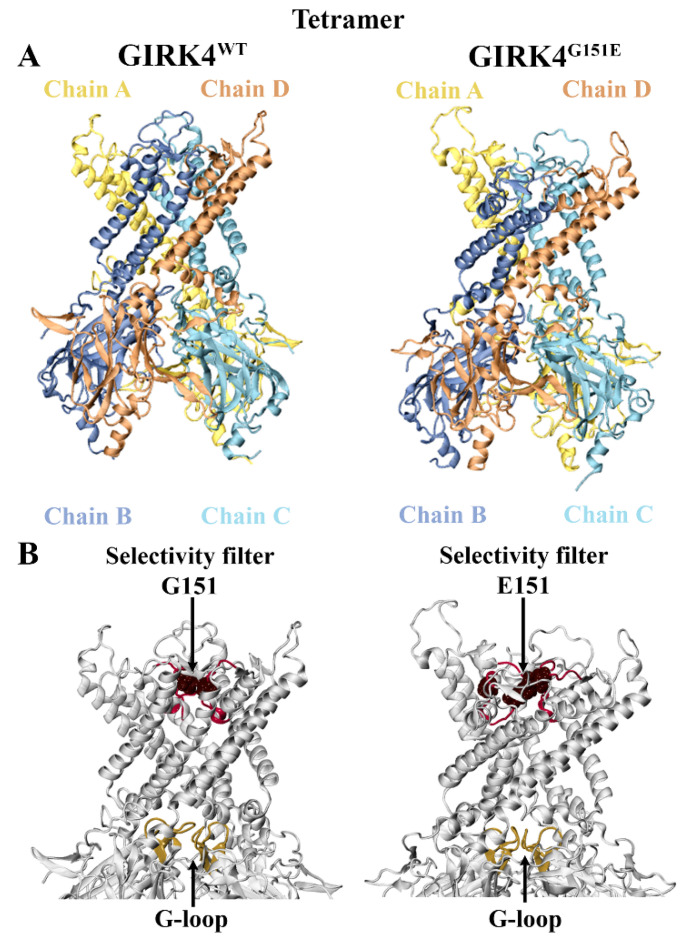
Homotetrameric structure of human G protein-gated inwardly rectifying K^+^ channel 4 (GIRK4). (**A**) Homology modeling was used to generate the wild-type (WT) and mutant (G151E) forms of GIRK4. (**B**) The G151E mutation is situated in the selectivity filter, which is comprised of residues T149-G153 (colored red). The G-loop region is located at the apex of the cytoplasmic domain and is colored brown.

Mammalian GIRK channels function as tetramers and have been found to exist as either homomeric or heteromeric structures [14,24]. The channel is composed of a pore-forming transmembrane domain (TMD) that contains a K^+^ selectivity filter and a cytoplasmic domain (CTD), which are covalently joined by the TMD-CTD linker [10,25]. The cytoplasmic pore and N- and C-termini are located within the CTD [10]. The selectivity filter is a highly conserved region among eukaryotic GIRK channels that is comprised of the sequence T-X-G-Y(F)-G [26,27]. Moreover, GIRK channels consist of the turret region, inner helix activation gate (helix bundle crossing), and G-loop gate at the apex of the cytoplasmic domain [10,26,28]. In this study, homology modeling and molecular dynamic (MD) simulations were used to obtain the structures of homotetrameric GIRK4^WT^ and GIRK4^G151E^ (Figure 1A). The pathogenic G151E mutation occurs within the conserved selectivity filter sequence and is highlighted in Figure 1B.

### 2.1. Protein–Peptide Docking of Tertiapin and Tertiapin-Q to Homotetrameric GIRK4

**Figure 2:**
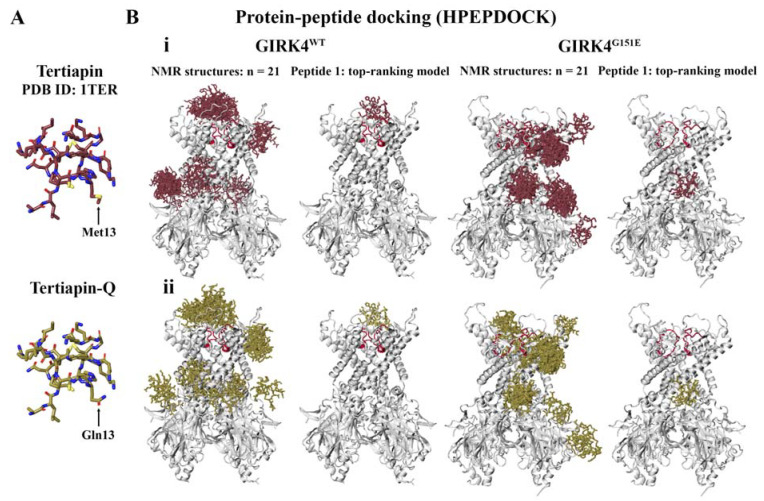
Flexible protein–peptide docking was performed to predict the binding site of known peptide inhibitors. (**A**) The structure of tertiapin was mutated at residue 13 to generate tertiapin-Q. (**B**) Blind protein–peptide docking was performed to evaluate the preferential binding site of tertiapin (**i**) and tertiapin-Q (**ii**) against human G protein-gated inwardly rectifying K^+^ channel 4 (GIRK4). This included the GIRK4^WT^ and GIRK4^G151E^ channels. All 21 NMR conformations of tertiapin were utilized, and the top-ranking model for each structure is shown. The protein–peptide docking results for the first structure of tertiapin (peptide 1) and tertiapin-Q provided in the PDB file are also shown. Tertiapin and tertiapin-Q were predicted to preferentially bind to the selectivity filter of GIRK4^WT^; however, the peptides were displaced for GIRK4^G151E^.

Previous studies have demonstrated that tertiapin, a 21-amino-acid peptide (sequence: A-L-C-N-C-N-R-I-I-I-P-H-M-C-W-K-K-C-G-K-K) derived from honey bee venom, inhibits certain types of inward rectifier K^+^ channels, including GIRK1/4 and ATP-sensitive inward rectifier potassium channel 1 (ROMK1) [29,30]. Tertiapin-Q is a stable oxidation-resistant derivative of tertiapin, whereby the methionine residue is mutated to glutamine (sequence: A-L-C-N-C-N-R-I-I-I-P-H-Q-C-W-K-K-C-G-K-K) (Figure 2A) [29,30]. The effects of tertiapin and tertiapin-Q on recombinant human BK-type K^+^ channels and acetylcholine-induced muscarinic K^+^ (K_ACh_) channels in rabbit cardiac myocytes have also been investigated [31,32].

Computational approaches have been employed to explore the binding mode of tertiapin against GIRK2 [33,34,35,36]. Using molecular docking and MD simulations, Patel et al. showed that the GIRK2 subunit is essential for the high-affinity binding of tertiapin to neuronal heteromeric GIRK channels [34]. For the homotetrameric GIRK2 channel, a lysine residue of tertiapin was found to protrude into the pore region, and interactions were detected with the selectivity filter motif [34]. The peptide inhibitor also interacted with the turret region [34]. The MD simulations indicated that for the homotetrameric GIRK1 channel, tertiapin interacted only with the selectivity filter and dissociated within 10 nanoseconds [34]. The instability of the tertiapin–GIRK1 complex was likely due to the absence of acidic amino acids in the turret region, which facilitate interactions with the basic residues of the peptide inhibitor [34]. Similar to the findings for GIRK2, Ramu et al. demonstrated that GIRK4 is sensitive to tertiapin-Q and the presence of the subunit in heterotetrametric GIRK1/4 channels confers the high affinity for the inhibitor [37]. Ramu et al. also identified a short region in the N-terminal part of the M1-M2 linker that is critical for the high-affinity binding of tertiapin-Q [37].

In the adrenal cortex, which contains the aldosterone-secreting zona glomerulosa cells, GIRK4 exists as homomeric channels [14,38]. In this study, blind protein–peptide docking was performed to characterize the binding site of tertiapin and its derivative on the homotetrameric GIRK4^WT^ channel. We also evaluated the potential effects of the pathogenic G151E mutation on the binding of tertiapin and tertiapin-Q. The NMR solution structure of tertiapin included 21 peptide conformers (Figure 2A) [39]. To generate the structure of tertiapin-Q, the methionine residue was manually mutated to glutamine (Figure 2A). When considering the highest scoring model for each conformer, the results revealed that the peptide inhibitors were preferentially binding to the selectivity filter region of the homotetrameric GIRK4^WT^ channel compared to the mutated GIRK4^G151E^ channel (Figure 2B).

The interactions and interface residues of the peptide conformers that were predicted to bind in proximity to the selectivity filter were analyzed using the PDBePISA server (Tables S1 and S2). The results revealed that for the GIRK4^WT^ channel, the tertiapin and tertiapin-Q conformers were either (1) interacting with the conserved selectivity filter sequence, including G151, Y152, and G153, or (2) interacting with both the selectivity filter sequence and the short D-L-D-H-V-G-D-Q-E-W-I-P-C-V sequence (residues D117-V130) identified by Ramu et al. [37].

The protein–peptide docking results for the first conformer in the NMR solution structure can be seen in Figure 2B. The PDBePISA server predicted tertiapin and tertiapin-Q to form hydrogen bonds with G153 (chain D) and Y152 (chain C), as well as a salt bridge with E131 (chain C). Residues T149-G153 correspond to the T-I-G-Y-G sequence of the GIRK4^WT^ selectivity filter [26]. The structures of tertiapin and tertiapin-Q were predicted to bind away from the selectivity filter region for the GIRK4^G151E^ channel and formed a hydrogen bond with H64 (chain D). Mutated GIRK4 channels have been found to exhibit a different pharmacological profile compared to the WT form and are less sensitive to the effects of tertiapin-Q [8,15].

Furthermore, naringin has been found to directly activate GIRK channels [18]. Yow et al. demonstrated that the activation of heterotetrametric GIRK1/GIRK4 channels by naringin could be competitively inhibited by tertiapin-Q, indicating a common or overlapping binding site that has been hypothesized to occur on the GIRK4 subunit [18]. Using the predicted binding site of tertiapin-Q from the protein–peptide docking analysis, molecular docking was performed to evaluate the interactions between naringin and the homotetrameric GIRK4 channels. The flavonoid glycoside was predicted to bind to the GIRK4^WT^ and GIRK4^G151E^ channels with an affinity of −7.7 kcal/mol. Naringin was predicted to form a hydrogen bond with G151 (chain C) and a π-cation with R155 (chain C), which are located within the selectivity filter of the GIRK4^WT^ homotetrameric channel. Naringin was also predicted to form a hydrogen bond with the mutated E151 residue (chain C) of the GIRK4^G151E^ homotetrameric channel.

In a study by Jin et al., the amino acids F146 and F148 in the M1-M2 linker of the ROMK1 channel were found to affect the affinity of tertiapin-Q [30]. By performing a sequence alignment, Yow et al. identified the corresponding residues to be Y148 and Y150 in the GIRK1 channel and highlighted the contribution of the amino acids to the binding or gating of naringin [18]. In the human GIRK4 sequence, residues Y148 and Y150 correspond to F154 and V156, respectively. When evaluating the protein–ligand interactions for the GIRK4^WT^ and GIRK4^G151E^ channels, the hydrophobic amino acids F154 and V156 were predicted to be located within 5 Å of naringin. Similarly, F154 and V156 were identified as interface residues for several of the tertiapin and tertiapin-Q conformers generated from protein–peptide docking (Tables S1 and S2).

### 2.2. Identification of Potential Ligand-Binding Sites in the GIRK4^WT^ and GIRK4^G151E^ Channels

**Figure 3:**
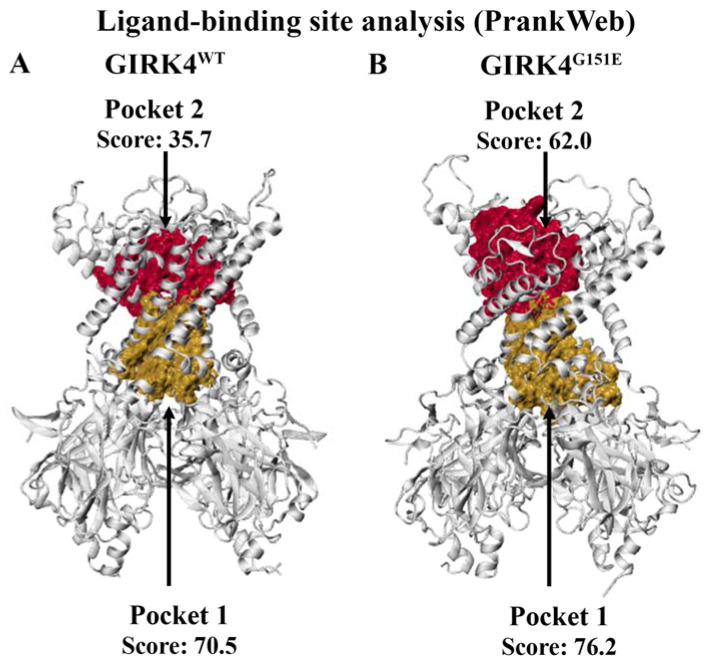
The PrankWeb server was used to predict potential ligand-binding sites for the structures of the wild-type (WT) and G151E mutant forms of the human G protein-gated inwardly rectifying K^+^ channel 4 (GIRK4). (**A**) The top-ranking sites for GIRK4^WT^ are depicted, with predicted ligandability scores of 70.5 and 35.7 for pocket 1 and pocket 2, respectively. (**B**) For GIRK4^G151E^, pocket 1 and pocket 2 had predicted ligandability scores of 76.2 and 62.0, respectively. Pocket 1 was found to encompass the G-loop region, while pocket 2 was comprised of the selectivity filter and central cavity region.

Prior to molecular docking, the PrankWeb server was used to predict potential ligand-binding sites in the GIRK4^WT^ and GIRK4^G151E^ proteins. The top-ranking pocket for both structures included residues that form the G-loop gate. In a study by Li et al., the G-loop gate of the Kir2.1 channel was reported to consist of residues M301 to T308 [40]. In the human GIRK4 sequence, the corresponding residues are M308 to T315. As seen in Figure 3, the ligandability scores for this region (pocket 1) in the GIRK4^WT^ and GIRK4^G151E^ channels were 70.5 and 76.2, respectively.

The second putative binding site predicted by PrankWeb was comprised of residues that form the conserved selectivity filter sequence and central cavity. The ligandability scores were calculated to be 35.7 and 62.0 for the GIRK4^WT^ and GIRK4^G151E^ structures, respectively. The residues forming the two highest scoring pockets identified from the PrankWeb analysis were used to define the target binding sites for molecular docking (Table S3).

### 2.3. Molecular Docking to the Central Cavity Region of Homotetrameric GIRK4

**Figure 4:**
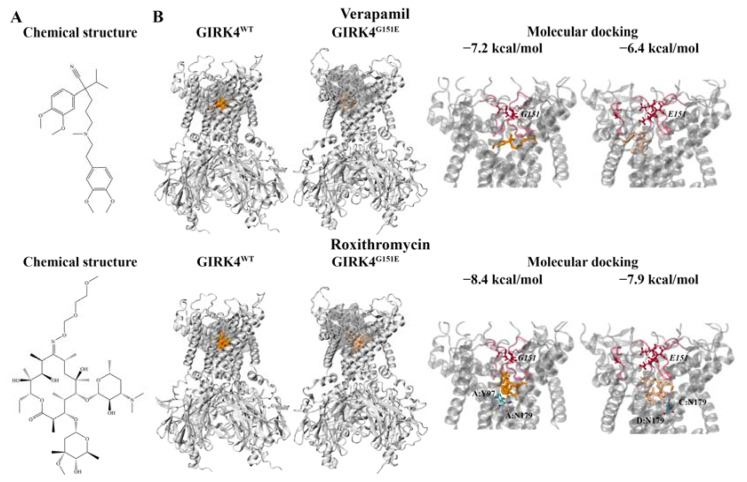
Molecular docking results for the compounds verapamil and roxithromycin. (**A**) The chemical structures of the calcium channel inhibitor verapamil and macrolide antibiotic roxithromycin are provided. (**B**) The position of the inhibitors within the pocket identified from the ligand-binding-site analysis can be seen. The inhibitors are colored orange and beige for the wild-type (WT) and mutated GIRK4 channels, respectively. The binding affinities (kcal/mol) are provided, along with the protein residues that were predicted to be involved in hydrogen bond interactions with the ligands. The G151 and mutated E151 residues are italicized. The selectivity filter motif is colored red.

Tauber et al. previously demonstrated the altered pharmacology of the GIRK4^L168R^ channel compared to GIRK4^WT^ [15]. The GIRK4^L168R^ channel was inhibited by various drugs targeting Na^+^ channels, Ca^2+^ channels, and Na^+/^Ca^2+^ exchangers [15]. This included verapamil (IC_50_ = 1.2 μM), EIPA (IC_50_ = 0.6 μM), KB-R7943 (IC_50_ = 0.8 μM), and nifedipine (IC_50_ = 53 μM) [15]. Additionally, verapamil inhibited the GIRK4^G151R^ and GIRK4^T158A^ mutant channels [15]. Roxithromycin has also been found to be a potent inhibitor of the GIRK4^G151R^ and GIRK4^L168R^ mutant channels [41]. The chemical structures of verapamil and roxithromycin are depicted in Figure 4A.

To investigate the binding characteristics of verapamil, EIPA, KB-R7943, nifedipine, and roxithromycin against the region encompassing the selectivity filter sequence and central cavity (pocket 2 from the PrankWeb analysis) of the GIRK4 channels, molecular docking was performed. Similar binding site residues (within 5 Å) were observed for roxithromycin, verapamil, EIPA, and KB-R7943 in the GIRK4^WT^ channel. The amino acids included Y97, W101, E147, T148, A172, G175, S176, and N179 from chain A and T146, L168, and L169 from chain C. KB-R7943 formed a hydrogen bond with S143 from chain C, while EIPA formed hydrogens bonds with Y97 and S176 from chain A (Table 1). Roxithromycin was predicted to form hydrogen bonds with N179 in both the GIRK4^WT^ and GIRK4^G151E^ channels (Figure 4B). Due to the relatively large size of roxithromycin within the predicted binding site, the orientation of the macrolide antibiotic also overlapped with that of nifedipine. Common residues within 5 Å included E147, T148, and T149 from chain B and E147, T148, T149, I150, A172, and S176 from chain D.

In eukaryotic Kir channels, the central region of the pore is comprised of four polar residues that project toward the ion pathway [26]. Tao et al. reported that these polar amino acids are D173 in the structure of Kir2.2 [26]. The corresponding residue in the human GIRK4 channel is N179. Furthermore, Cui et al. showed that BP-G1 inhibited the GIRK1/GIRK4 channel through binding to the central pore cavity, preventing ions from passing through [42]. The MD simulations also revealed that BP-G1 may stabilize the closed state of the G-loop gate through allosteric mechanisms [42]. Cui et al. performed molecular docking to evaluate the interactions of BP-G1 with the GIRK4/GIRK4(S143F) heteromers [42]. The residues forming the binding site included Y97, E147, T148, T149, A172, G175, and N179 [42]. This is in accordance with our findings, which demonstrate that the compounds bind in proximity to the conserved selectivity filter motif and are positioned in the central cavity of the GIRK4^WT^ and GIRK4^G151E^ channels (Figure 4B). This region is distinct from the binding site of tertiapin peptide inhibitors and naringin.

For the mutated GIRK4^G151E^ channel, the orientation of verapamil, EIPA, and nifedipine within the binding site overlapped with roxithromycin. The surrounding residues included E147, T149, and S176 from chain A and T149, A172, and G175 from chain B. Nifedipine was predicted to form a hydrogen bond with S176 of chain B, while EIPA formed hydrogen bonds with Y97 and E147 of chain B (Table 1). The common binding site residues for roxithromycin and KB-R7943 included T146, E147, T148, T149, S176, and N179 from chain C and A172, G175, S176, and N179 from chain D. Similar to roxithromycin, KB-R7943 was predicted to form a hydrogen bond with N179 of chain C. KB-R7943 also formed hydrogen bonds with W108 and E147 of chain C (Table 1).

Several studies have investigated the therapeutic effects of natural compounds, including flavonoids, on Kir channels [43]. To identify potential novel lead compounds with modulatory activity, we screened the OliveNet^TM^ library against the central cavity region of the GIRK4^WT^ and GIRK4^G151E^ channels [44]. Antimicrobial agents, including macrolide antibiotics, from the EpiMed Coronabank Chemical Collection were also utilized [45]. In total, 797 compounds were utilized, and the full list has been provided in the Supplementary Information (Tables S4 and S5). The binding affinities of the molecules ranged from −2.2 to −10.7 kcal/mol and −2.2 to −10.2 kcal/mol for GIRK4^WT^ and GIRK4^G151E^, respectively (Table S4). Most notably, the xanthophylls from the OliveNet^TM^ library were predicted to preferentially bind to the GIRK4^G151E^ channel compared to the GIRK4^WT^ channel. Luteoxanthin had a predicted binding affinity of −9.6 kcal/mol for the GIRK4^G151E^ channel. Hydrogen bonds were detected with residue N179 from chain A and R155 from chain B.

**Figure 5:**
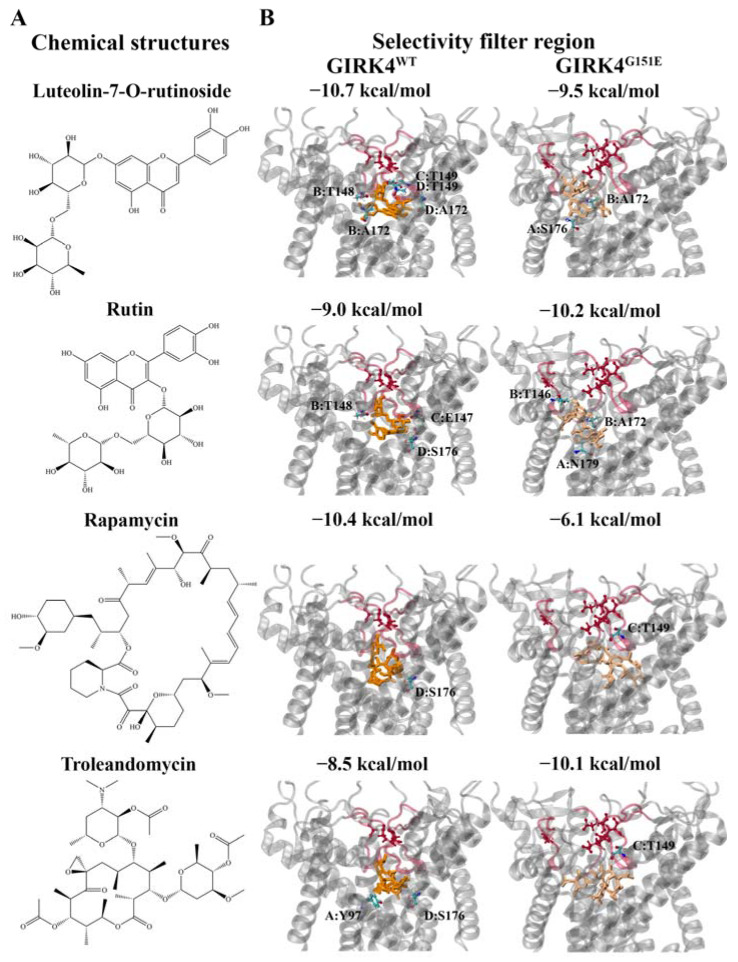
Molecular docking results for the potential lead compounds in the central cavity region of the wild-type (WT) and G151E mutant forms of the human G protein-gated inwardly rectifying K^+^ channel 4 (GIRK4) channels. (**A**) The chemical structures of the flavonoids from the OliveNet^TM^ library, luteolin-7-O-rutinoside and rutin, and macrolides, rapamycin and troleandomycin, are provided. (**B**) The binding affinities (kcal/mol) are provided, along with the protein residues that were predicted to be involved in hydrogen bond interactions with the ligands. The selectivity filter motif is colored red. The inhibitors are colored orange and beige for the WT and mutated GIRK4 channels, respectively.

Luteolin-7-O-rutinoside (−10.7 kcal/mol) and rapamycin (−10.4 kcal/mol) were predicted to be the strongest binding ligands for the central cavity region of the GIRK4^WT^ channel (Figure 5A). The flavonoid luteolin-7-O-rutinoside was predicted to form hydrogen bonds with A172 and T148 from chain B, T149 from chain C, and T149 and A172 from chain D (Figure 5B). A hydrogen bond with S176 from chain D was detected for the macrolide rapamycin (Figure 5B). We also examined the protein–ligand interactions of the strongest binding flavonoid and macrolide for the GIRK4^G151E^ channel. Rutin and troleandomycin were predicted to have binding affinities of −10.2 and −10.1 kcal/mol, respectively (Figure 5A). Rutin formed hydrogen bonds with N179 from chain A, T146 from chain B, and A172 from chain B (Figure 5B). Troleandomycin was observed to form a hydrogen bond with T149 from chain C (Figure 5B).

### 2.4. Molecular Docking to the Region Encompassing the G-Loop Gate

Additionally, the putative binding site (pocket 1 from the PrankWeb analysis) encompassing the G-loop gate was of interest. In a study by Trezza et al., the inhibitory activity of the flavonoid quercetin against the *Rattus norvegicus* Kir6.1 channel was evaluated, and the G-loop region was the focus of the computational analysis [46]. Using molecular docking, quercetin was predicted to form several hydrogen bonds and hydrophobic interactions with residues in this site [46].

Our results revealed that the binding affinities of the 797 compounds for the G-loop region of the GIRK4^WT^ and GIRK4^G151E^ channels ranged from −0.1 to −9.8 kcal/mol and −1.2 to −11.0 kcal/mol, respectively (Table S5). Interestingly, the triterpene alcohols and triterpenic acids from the OliveNet^TM^ database were predicted to bind with a stronger affinity to the G-loop region of the mutated GIRK4^G151E^ channel compared to the GIRK4^WT^ form.

**Figure 6:**
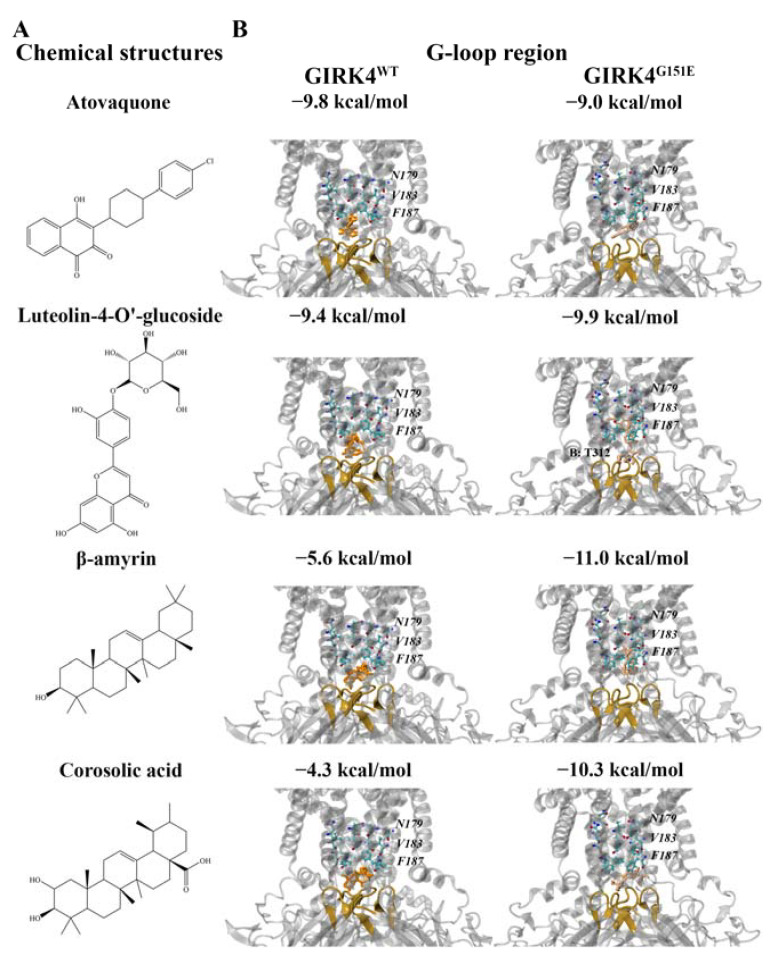
Molecular docking results for the G-loop region of the wild-type (WT) and G151E mutant forms of the human G protein-gated inwardly rectifying K^+^ channel 4 (GIRK4) channels. (**A**) The chemical structures of atovaquone (antimicrobial agent), luteolin-4′-O-glucoside (flavonoid), β-amyrin (triterpene alcohol), and corosolic acid (triterpenic acid) are provided. (**B**) The binding affinities (kcal/mol) are provided, along with the protein residues that were predicted to be involved in hydrogen bond interactions with the ligands. Key residues N179, V183, and F187 are italicized. The inhibitors are colored orange and beige for the WT and mutated GIRK4 channels, respectively. The G-loop gate is colored brown.

The protein–ligand interactions of atovaquone (antimicrobial agent), luteolin-4′-O-glucoside (flavonoid), β-amyrin (triterpene alcohol), and corosolic acid (triterpenic acid) were analyzed further (Figure 6A). Atovaquone (−9.8 kcal/mol) and luteolin-4′-O-glucoside (−9.4 kcal/mol) were predicted to be the strongest binding ligands for the G-loop region of the GIRK4^WT^ channel. The key residues within 5 Å of the ligands in the GIRK4^WT^ channel included G184, F187, and V188 from chain A, F187, V188, T312, and G313 from chain B, F187, V188, S191, A311, T312, and G313 from chain C, and F78, F187, V188, S191, Q192, P193, and T312 from chain D.

Β-amyrin (−11.0 kcal/mol) and corosolic acid (−10.3 kcal/mol) were predicted to be the strongest binding triterpene alcohol and triterpenic acid, respectively, for the G-loop region of the mutated GIRK4^G151E^ channel. For the GIRK4^G151E^ channel, the key residues were F187, T312, and M314 for chain A, F187, V188, T312, and M314 for chain B, F187, V188, and M314 for chain C, and F187 for chain D. As seen in Figure 6B, luteolin-4′-O-glucoside was predicted to form a hydrogen bond with T312 from chain B. Taken together, the compounds were predicted to bind in close proximity to the G-loop gate and were also surrounded by residues of the helix bundle crossing, including F187.

## 3. Materials and Methods

### 3.1. Preparation of Protein Structures

A homology model of the homotetrameric GIRK4^WT^ channel was generated using the SWISS-MODEL server [47]. The amino acid sequence of the *KCNJ5* gene was obtained from the UniProt database (ID: P48544) [48]. The cryo-EM structure of the mouse GIRK2 channel (PDB ID: 6XIT) was used as the template (3.3 Å resolution, 83.13% sequence identity) [25]. The stereochemical quality of the model was analyzed using PROCHECK, with 91.5%, 8.1%, and 0.3% of residues belonging to most favored, additionally allowed, and generously allowed regions of the Ramachandran plot, respectively (Figure S1) [49].

Four molecules of the co-crystallized ligands from the template bound to the channel were modified to obtain coordinates of the cofactor phosphatidylinositol 4,5-bisphosphate (PIP_2_). Since there was a high sequence similarity (83.13%) and overlap between the template and homology model (RMSD = 2.31 Å), crystallographic cofactors were utilized as a starting point to generate the GIRK4 tetramer bound with four molecules of PIP_2_. PIP_2_ acyl chains were only partially resolved in the crystal structure [25]. The co-ordinates of partially resolved crystal cofactors for each chain were modified to generate full PIP_2_ molecules and topologies using the Ligand Reader & Modeler in CHARMM-GUI [50,51]. The system then underwent molecular dynamic (MD) simulations to relax the tetrameric model and cofactors to obtain a stable structure for docking.

MD simulations were performed as previously described [52] with Gromacs 2018.2 and the CHARMM36 force field [53,54,55]. The topology of PIP_2_ was obtained using the CHARMM General Force Field (CGenFF) Program v4.6. Coordinates of GIRK4^WT^ and GIRK4^G151E^ in the final frame of the production run (200 ns, 2 fs time step) were used for molecular docking. The structures of the homotetrameric GIRK4^WT^ and GIRK4^G151E^ channels were prepared as macromolecules in AutoDockTools-1.5.7 [56].

### 3.2. Preparation of Ligands

Several GIRK4 channel inhibitors were identified from the literature and were used as control compounds [15,41]. This included verapamil, ethylisopropylamiloride (EIPA), KB-R7943, nifedipine, and roxithromycin [15,41]. Flavonoids and macrolide antibiotics that have been reported to modulate the activity of Kir channels were also of interest [18]. Moreover, the natural compounds from the OliveNet^TM^ library and antimicrobial agents from the EpiMed Coronabank Chemical Collection were utilized [44,45]. The chemical structures of the compounds were obtained from the National Center for Biotechnology Information PubChem database [57]. If the three-dimensional (3D) structures were unavailable, the two-dimensional structures were downloaded and converted to the 3D format using Chem3D (v21.0.0.28, PerkinElmer Informatics). The molecules were imported into PyRx, they were energy minimized using the universal force field through Open Babel (v.2.2.3), and they were prepared as ligands [58,59].

### 3.3. Ligand-Binding Site Analysis and Molecular Docking

The structures of the GIRK4^WT^ and GIRK4^G151E^ channels were uploaded to the PrankWeb server to identify potential ligand-binding sites using conservation analysis [60]. The compounds were subsequently screened against the putative ligand-binding sites identified by the PrankWeb server. This included the selectivity filter and central cavity region (pocket 2), as well as the G-loop region (pocket 1) of the GIRK4^WT^ and GIRK4^G151E^ channels. The receptor grids were generated using the residues that were predicted to form part of each target site and were 25 × 25 × 25 Å in size. Molecular docking was performed using AutoDock Vina at an exhaustiveness of 128 [61]. Maestro 13.2 and Visual Molecular Dynamics 1.9.3 were used to analyze the results [62,63].

### 3.4. Protein–Peptide Docking

Protein–peptide docking was performed to investigate the interaction of tertiapin and tertiapin-Q, which inhibit Kir channel isoforms, with the homotetrameric GIRK4^WT^ and GIRK4^G151E^ channels. The nuclear magnetic resonance (NMR) structure of tertiapin was obtained from the RCSB Protein Data Bank (PDB ID: 1TER), with all 21 solution structures being utilized [39]. The tertiapin-Q derivative was generated by mutating the methionine amino acid at position 13 to glutamine using PyMOL [29,64]. The HPEPDOCK 2.0 server was used to perform blind protein–peptide docking, and the flexibility of the peptides was considered [65]. The Proteins, Interfaces, Structures and Assemblies (PDBePISA) server was used to evaluate the predicted interactions (hydrogen bonds and salt bridges) and interface residues for the top-ranking model of each peptide conformer [66]. Molecular docking was performed using AutoDock Vina (exhaustiveness = 128) to evaluate and compare the interactions of naringin with the predicted binding site of tertiapin-Q for the GIRK4^WT^ (X: 53.42, Y: 48.51, X: 115.78) and GIRK4^G151E^ (X: 44.93, Y: 57.58, Z: 115.43) channels [61]. The receptor grids were 25 × 25 × 25 Å in size.

## 4. Conclusions

Overall, we employed *in silico* tools to generate a homology model of the human homotetrameric GIRK4^WT^ channel and the pathogenic GIRK4^G151E^ variant. Based on the ligand-binding site analysis, which took into consideration evolutionary conservation, two major binding pockets were identified. Flexible protein–peptide docking was performed to confirm the interaction of control tertiapin peptides with the selectivity filter motif. Moreover, the central cavity region was identified as the key site for the binding of small molecules, particularly the xanthophylls and flavonoids for the mutated GIRK4^G151E^ channel, as well as the macrolides roxithromycin and troleandomycin, which had high affinities for the analogous region. The triterpene alcohols and triterpenic acids, including β-amyrin and corosolic acid, had the highest binding affinities for the putative binding site, encompassing the G-loop gate in the mutated GIRK4^G151E^ channel. More generally, we have further defined potential binding sites associated with the homotetrameric GIRK4 channels and have identified interesting compounds that interact with these regions. The protein–ligand complexes can be further analyzed using MD simulations, and the modulatory activity of these compounds can be examined *in vitro* and *in vivo*.

## Figures and Tables

**Table 1:** Binding affinities (kcal/mol) and protein–ligand interactions for the central cavity of the GIRK4^WT^ and GIRK4^G151E^ channels.

	GIRK4^WT^		GIRK4^G151E^	
Compound	Binding Affinity (kcal/mol)	Interactions	Binding Affinity (kcal/mol)	Interactions
EIPA	−6.7	A: Y97 (H-bond), S176 (H-bond)	−6.3	B: Y97 (H-bond), E147 (H-bond)
KB-R7943	−7.6	C: S143 (H-bond)	−7.4	C: W108 (H-bond), E147 (H-bond), N179 (H-bond)
Nifedipine	−6.1	-	−6.1	B: S176 (H-bond)
Verapamil	−7.2	-	−6.4	-
Roxithromycin	−8.4	A: Y97 (H-bond), N179 (H-bond)	−7.9	C: N179 (H-bond), D: N179 (H-bond)

## Data Availability

The data presented in this study are available on request from the corresponding author.

## Supplementary Materials

The following supporting information can be downloaded at: https://www.mdpi.com/article/10.3390/molecules28247946/s1, Figure S1: The stereochemical quality of the human GIRK4^WT^ homology model was assessed using PROCHECK; Table S1: Interactions and interface residues between the GIRK4^WT^ channel and top-ranking model of the tertiapin peptide conformers that were predicted to bind in proximity to the selectivity filter region; Table S2: Interactions and interface residues between the GIRK4 channels and top-ranking model of the tertiapin-Q peptide conformers that were predicted to bind in proximity to the selectivity filter region; Table S3: Putative ligand-binding sites identified from the PrankWeb analysis for the homotetrameric GIRK4^WT^ and GIRK4^G151E^ channels; Table S4: Predicted binding affinities (kcal/mol) of the compounds screened against the central cavity region of the GIRK4^WT^ and GIRK4^G151E^ channels. Table S5: Predicted binding affinities (kcal/mol) of the compounds screened against the G-loop region of the GIRK4^WT^ and GIRK4^G151E^ channels.
