# Supplementary material for: Identification of Potential Modulators of a Pathogenic G Protein-Gated Inwardly Rectifying K+ Channel 4 Mutant: In Silico Investigation in the Context of Drug Discovery for Hypertension
Source: Molecules. 2023 Dec 5;28(24):7946. doi: 10.3390/molecules28247946 (PMC10745636; doi:10.3390/molecules28247946)
Supplement: Supplementary file 1 [file molecules-28-07946-s001.zip › molecules-2738823-supplementary.pdf]

Supplementary Information

Article

Identification of Potential Modulators of a Pathogenic G Protein-Gated Inwardly Rectifying K^+ Channel 4 Mutant: *In Silico* Investigation in the Context of Drug Discovery for Hypertension

Eleni Pitsillou [1,2], Alexander N. O. Logothetis [1,3], Julia J. Liang [1,2,4], Assam El-Osta [4,5,6,7,8,9], Andrew Hung [2], Asmaa S. AbuMaziad [10] and Tom C. Karagiannis [1,3,4,11,*]

[1] Epigenomic Medicine Laboratory at prospED Polytechnic, Carlton, VIC 3053, Australia
[2] School of Science, STEM College, RMIT University, Melbourne, VIC 3001, Australia
[3] Department of Microbiology and Immunology, The University of Melbourne, Parkville, VIC 3010, Australia
[4] Epigenetics in Human Health and Disease Program, Baker Heart and Diabetes Institute, 75 Commercial Road, Prahran, VIC 3004, Australia
[5] Department of Diabetes, Central Clinical School, Monash University, Melbourne, VIC 3004, Australia
[6] Department of Medicine and Therapeutics, The Chinese University of Hong Kong, Sha Tin, Hong Kong SAR, China
[7] Hong Kong Institute of Diabetes and Obesity, Prince of Wales Hospital, The Chinese University of Hong Kong, 3/F Lui Che Woo Clinical Sciences Building, 30-32 Ngan Shing Street, Sha Tin, Hong Kong SAR, China
[8] Li Ka Shing Institute of Health Sciences, The Chinese University of Hong Kong, Sha Tin, Hong Kong SAR, China
[9] Biomedical Laboratory Science, Department of Technology, Faculty of Health, University College Copenhagen, 1799 Copenhagen, Denmark
[10] Department of Pediatrics, College of Medicine Tucson, The University of Arizona, Tucson, AZ 85724, USA 11 Department of Clinical Pathology, The University of Melbourne, Parkville, VIC 3010, Australia
[*] Correspondence: karat@unimelb.edu.au; Tel.: +613-8532-1290; Fax: +613-8532-1100

Contents

Figure S1. The stereochemical quality of the human GIRK4^{WT} homology model was assessed using PROCHECK.

Table S1. Interactions and interface residues between the GIRK4^{WT} channel and top-ranking model of the tertiapin peptide conformers that were predicted to bind in proximity to the selectivity filter region.

Table S2. Interactions and interface residues between the GIRK4 channels and top-ranking model of the tertiapin-Q peptide conformers that were predicted to bind in proximity to the selectivity filter region.

Table S3. Putative ligand-binding sites identified from the PrankWeb analysis for the homotetrameric GIRK4^{WT} and GIRK4^{G151E} channels.

Table S4. Predicted binding affinities (kcal/mol) of the compounds screened against the central cavity region of the GIRK4^{WT} and GIRK4^{G151E} channels.

Table S5. Predicted binding affinities (kcal/mol) of the compounds screened against the G-loop region of the GIRK4^{WT} and GIRK4^{G151E} channels.

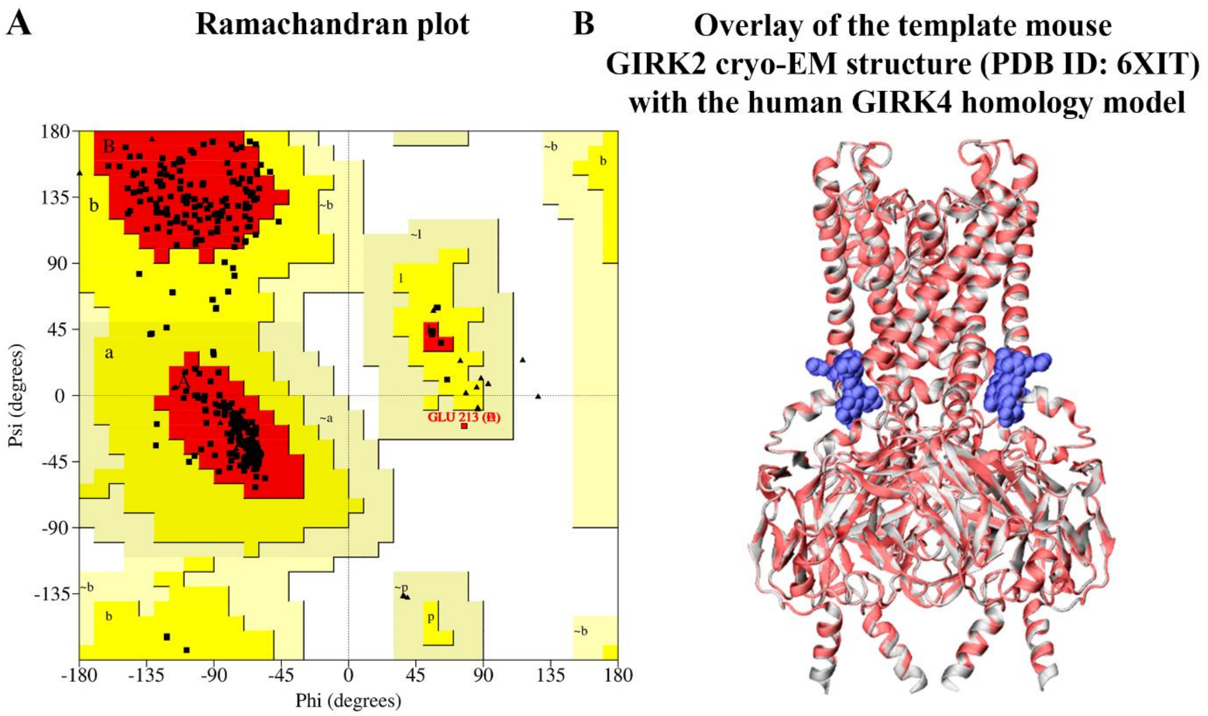

Figure S1. The stereochemical quality of the human GIRK4^{WT} homology model was assessed using PROCHECK. (A) The Ramachandran plot is shown with 91.5% of residues being located in the most favored regions, 8.1% of residues being located in the additional allowed regions, and 0.3% of residues being located in the generously allowed regions. (B) An overlay of the cryo-EM structure of the mouse GIRK2 channel (colored silver) that was used as the template and the homology model of the human GIRK4 homotetrameric channel (colored red) in complex with four molecules of the phosphatidylinositol 4,5-bisphosphate (PIP_2) cofactor is shown. The root-mean-square deviation (RMSD) was found to be 2.31 Å.

Table S1. Interactions and interface residues between the GIRK4^{WT} channel and top-ranking model of the tertiapin peptide conformers that were predicted to bind in proximity to the selectivity filter region.

Peptide conformer	Interactions	Protein interface residues	Peptide interface residues
**Peptide 1**	Chain A: none	G151, Y152, G153, F154, V156	L2, C3, C14, K17
	Chain B: none	N132, G151, Y152, G153, F154, V156	K16, K17, C18, G19
	Chain C: C5 – Y152 (H-bond), A1 – E131 (salt bridge)	E131, N132, Y152, G153, F154, V156, T158	A1, L2, C3, N4, C5, N6, R7
	Chain D: C5 – G153 (H-bond), C18 – G153 (H-bond)	E131, N132, Y152, G153, F154, V156, I157, T158, E159	C5, N6, R7, I8, C18, G19, K20
**Peptide 2**	Chain A: A1 - G153 (H-bond)	G151, Y152, G153, F154, V156	A1, L2, M13
	Chain B: A1 - Y152 (H-bond)	P128, E131, N132, G151, Y152, G153, F154, V156	A1, L2, N4, R7, I8, I9, P11
	Chain C: none	Y152	M13
	Chain D: H12 - G153 (H-bond)	E131, N132, Y152, G153, F154, R155, V156	I9, I10, P11, H12, W15, K16, K21
		-	NH2 interface with F154
**Peptide 8**	Chain A: none	-	-
	Chain B: N4 - E131 (H-bond), R7 - E131 (H-bond), C18 - Q124 (H-bond), R7 - E131 (salt bridge)	D117, L118, H120, V121, G122, D123, Q124, E125, W126, I127, P128, E131, N132, L133, S134, V156	A1, L2, C3, N4, C5, N6, R7, M13, C14, K17, C18, G19
	Chain C: none	-	-
	Chain D: none	F154	N6, R7
**Peptide 11**	Chain A: N4 - E131 (H-bond), N6 - F154 (H-bond), R7 - E131 (H-bond), R7 - E131 (salt bridges x 4)	Q124, I127, P128, E131, G153, F154, R155, V156, I157, T158, E159, K160	A1, L2, C3, N4, C5, N6, R7
	Chain B: K17 - E131 (H-bond), C18 - G153 (H-bond), K17 - E131 (salt bridges x 2)	E131, N132, S134, S138, Y152, G153, F154, R155, V156	L2, C3, C5, I10, M13, C14, K17, C18
	Chain C: none	-	-
	Chain D: none	F154	K16, K17

**Peptide 15**	Chain A: C3 - Y152 (H-bond), N4 - G153 (H-bond), C5 - G153 (H-bond)	E131, N132, G151, Y152, G153, F154, R155, V156	C3, N4, C5, N6, R7, K17, C18
	Chain B: L2 - G153 (H-bond)	N132, G151, Y152, G153, F154, V156	A1, L2, C3, N4, R7
	Chain C: none	E131, N132, G151, Y152, G153, F154, V156	L2, C3, M13, K17
	Chain D: none	G151, Y152, G153, F154, V156	A1, L2, M13
**Peptide 16**	Chain A: none	-	-
	Chain B: A1 - V130 (H-bond), H12 - E131 (H-bond x 2), M13 - E131 (H-bond), C14 - E131 (H-bond x 2), M13 - N132 (H-bond), H12 - E131 (salt bridges x 2)	A112, D117, L118, D119, H120, V121, G122, D123, Q124, E125, W126, I127, P128, V130, E131, N132, L133, S134, G153, F154, V156, I157, T158, E159	A1, L2, C3, N4, R7, I8, I9, I10, P11, H12, M13, C14, W15
	Chain C: none	-	-
	Chain D: none	F154	H12, M13, K16
**Peptide 20**	Chain A: I9 - E131 (H-bond)	Q124, I127, P128, C129, V130, E131, G153, F154, R155, V156, I157, T158, E159, K160	N4, R7, I8, I10, P11, H12, W15, K21
	Chain B: C3 - N132 (H-bond), C14 - N132 (H-bond), L2 - G153 (H-bond)	E131, N132, L133, S134, G151, Y152, G153, F154, R155, V156	A1, L2, C3, N4, R7, I9, I10, P11, H12, M13, C14
	Chain C: none	-	-
	Chain D: none	Y152, G153, F154	A1, L2
			NH2 interface with Q124, I127
**Peptide 21**	Chain A: R7 - Q124 (H-bond), H12 - G153 (H-bond), H12 - F154 (H-bond), C14 - F154 (H-bond), N6 - Q124 (H-bond), A1 - E145 (salt bridge)	V121, G122, D123, Q124, W126, I127, P128, E131, N132, E145, G151, Y152, G153, F154, R155, V156	A1, L2, C3, N4, N6, R7, I8, I9, I10, P11, H12, M13, C14, W15
	Chain B: none	G151, Y152, G153, F154	H12, W15, K20, K21
	Chain C: A1 - E159 (H-bonds), A1 - E159 (salt bridges)	Y152, G153, E159, K160	A1, L2, M13
	Chain D: none	-	-

Table S2. Interactions and interface residues between the GIRK4 channels and top-ranking model of the tertiapin-Q peptide conformers that were predicted to bind in proximity to the selectivity filter region.

	**$GIRK4^{WT}$**		
**Peptide conformer**	**Interactions**	**Protein interface residues**	**Peptide interface residues**
**Peptide 1**	Chain A: none	G151, Y152, G153, F154, V156	L2, C3, C14, K17
	Chain B: none	N132, G151, Y152, G153, F154, V156	K16, K17, C18, G19
	Chain C: C5 – Y152 (H-bond), A1 – E131 (salt bridge)	E131, N132, Y152, G153, F154, V156, T158	A1, L2, C3, N4, C5, N6, R7
	Chain D: C5 – G153 (salt bridge), C18 – G153 (salt bridge)	E131, N132, Y152, G153, F154, V156, I157, T158, E159	C5, N6, R7, I8, C18, G19, K20
**Peptide 2**	Chain A: C3- E159 (H-bond)	I127, V156, I157, T158, E159, K160	A1, L2, C3, Q13, K17
	Chain B: C3 - S134 (H-bond), C5 - D117 (H-bond), C18 - S134 (H-bond), K17 - G135 (H-bond), C18 - D119 (H-bond)	W109, D117, L118, D119, H120, W126, P128, E131, N132, L133, S134, G135, S138, L141, Y152, F154, R155	A1, L2, C3, N4, C5, N6, R7, K17, C18, G19, K20
	Chain C: none	-	-
	Chain D: none	-	-
**Peptide 7**	Chain A: none	-	-
	Chain B: R7 - E131 (H-bond), R7 - N132 (H-bond), R7 - E131 (salt bridges x 2)	D117, H120, V121, G122, D123, Q124, E125, W126, I127, P128, E131, N132, L133	C5, N6, R7, I8, I9, C18, K20, K21
	Chain C: none	-	-
	Chain D: none	-	-
		-	NH2 interface with Q124 and E125
**Peptide 8**	Chain A: C3 - F154 (H-bond), Q13 - E131 (H-bond), K17 - E131 (H-bond), K17 - E131 (salt bridges x 2)	V121, G122, D123, Q124, E131, N132, S134, S138, L141, E145, Y152, F154, R155, V156	A1, L2, C3, N4, C5, N6, P11, H12, Q13, C14, K16, K17, C18
	Chain B: none	-	-
	Chain C: R7 - E131 (H-bond and salt bridge)	Q124, I127, E131, V156, T158, E159, K160	A1, L2, C3, N4, N6, R7, I8, I9
	Chain D: none	-	-

**Peptide 11**	Chain A: N4 - E131 (H-bond), N6 - F154 (H-bond), R7 - E131 (H-bond), R7 - E131 (salt bridges x 4)	Q124, I127, P128, E131, G153, F154, R155, V156, I157, T158, E159, K160	A1, L2, C3, N4, C5, N6, R7
	Chain B: Q13 - N132 (H-bond), C18 - G153 (H-bond), K17 - E131 (salt bridges)	P128, E131, N132, S134, S138, Y152, G153, F154, R155, V156	L2, C3, C5, Q13, C14, K17, C18
	Chain C: none	-	-
	Chain D: none	F154	K16, K17
**Peptide 13**	Chain A: R7 - G151 (H-bond), R7 - Y152 (H-bond)	G151, Y152, G153, F154, V156	N6, R7, I9
	Chain B: R7 - G151 (H-bond)	G151, Y152, G153, F154	N4, N6, R7
	Chain C: none	E131, N132, G151, Y152, G153, F154, V156	C5, N6, R7, I8, I9, K21
	Chain D: C5 - G153 (H-bond), N6 - Y152 (H-bond), K20 - E131 (H-bond), C18 - N132 (H-bond), K20 - E131 (salt bridge)	E131, N132, G151, Y152, G153, F154, V156, I157, T158	C5, N6, I8, K17, C18, G19, K20
		-	NH2 interface with F154
**Peptide 16**	Chain A: none	-	-
	Chain B: P11 - W126 (H-bond), H12 - E131 (H-bond), Q13 - F154 (H-bond), C14 - E131 (H-bond), H12 - E131 (salt bridges x 2)	D117, L118, D119, H120, V121, G122, D123, Q124, E125, W126, I127, P128, V130, E131, N132, L133, S134, G153, F154, V156, I157, T158, E159	A1, L2, C3, N4, R7, I8, I9, I10, P11, H12, Q13, C14, W15
	Chain C: none	-	-
	Chain D: none	F154	H12, Q13, K16
**Peptide 21**	Chain A: R7 - Q124 (H-bond), P11 - E131 (H-bond), H12 - G153 (H-bond), H12 - F154 (H-bond), C14 - F154 (H-bond), L2 - N132 (H-bond), N6 - Q124 (H-bond), A1 - E145 (salt bridge)	V121, G122, D123, Q124, W126, I127, P128, E131, N132, L141, E145, G151, Y152, G153, F154, R155, V156	A1, L2, C3, N4, N6, R7, I8, I9, I10, P11, H12, Q13, C14, W15
	Chain B: none	G151, Y152, G153, F154	H12, W15, K20, K21

	Chain C: A1 - E159 (H-bonds x 2, salt bridges x 2)	G153, E159, K160	A1, L2, Q13
	Chain D: none	-	-
	**GIRK4^{G151E}**		
**Peptide 12**	Chain A: K21 - E131 (salt bridges x 2)	E131, N132, G153, F154, R155, V156, I157, T158, E159	C5, N6, R7, I8, I9, W15, C18, K20, K21
	Chain B: A1 - I157 (H-bond)	E151, Y152, G153, R155, V156, I157, T158, E159	A1, L2, I9, I10, P11, W15, K21
	Chain C: R7 - E151 (salt bridges x 2)	I150, E151, Y152	N6, R7, I9
	Chain D: R7 - T149 (H-bond), N4 - E151 (H-bond), L2 - E151 (H-bond), A1 - E151 (salt bridges x 2)	T149, I150, E151, Y152, G153	A1, L2, C3, N4, N6, R7
	NH2 interface with E131, V156	-	-
**Peptide 19**	Chain A: L2 - E145 (H-bond), A1 - E145 (salt bridges x 2), H12 - E131 (salt bridges x 2), K17 - E159 (salt bridges x 2)	E131, N132, F142, E145, E151, G153, F154, R155, V156, I157, T158, E159, I165, L168	A1, L2, C3, I10, P11, H12, Q13, C14, K17
	Chain B: N6 - T146 (H-bond), C3 - T149 (H-bond), L2 - T149 (H-bond), C18 - Y152 (H-bond)	F142, T146, T148, T149, I150, E151, Y152, G153, F154, R155, V156, I157, L168	A1, L2, C3, N4, C5, N6, R7, I8, Q13, C14, K17, C18
	Chain C: none	E151	R7
	Chain D: I8 - E151 (H-bond), R7 - E151 (salt bridge)	T148, T149, I150, E151, Y152, G153	C5, N6, R7, I8, I9

Table S3. Putative ligand-binding sites identified from the PrankWeb analysis for the homotetrameric GIRK4^{WT} and GIRK4^{G151E} channels.

Channel	Ligand-binding site	Residues
**GIRK4^{WT}**	**Pocket 1**	Chain A: 176, 179, 180, 183, 184, 185, 187, 188, 311, 312, 314 78<br>Chain B: 179, 180, 183, 184, 187, 188, 191, 311, 312, 313, 314, 78, 79<br>Chain C: 179, 183, 184, 187, 188, 191, 311. 312, 314<br>Chain D: 176, 179, 180, 183, 184, 187, 188, 191, 192, 193, 312, 314, 78
	**Pocket 2**	Chain A: 100, 101, 104, 146, 147, 148, 149, 150, 151, 168, 171, 172, 175, 176, 178, 179, 182, 97<br>Chain B: 147, 148, 149, 150<br>Chain C: 101, 104, 108, 143, 146, 147, 148, 149, 151, 168, 169, 171, 172, 175, 176, 179, 97<br>Chain D: 147, 148, 149, 150, 172, 173, 176, 179
**GIRK4^{G151E}**	**Pocket 1**	Chain A: 176, 179, 180, 183, 184, 187, 188, 191, 192, 193, 194, 196, 197, 225, 227, 309, 311, 312, 313, 314, 315, 316, 317<br>Chain B: 176, 179, 180, 183, 184, 187, 188, 191, 310, 311, 312, 313, 314<br>Chain C: 176, 179, 180, 183, 184, 187, 188, 311, 312, 314, 78, 79, 82, 83<br>Chain D: 179, 180, 183, 184, 187, 188, 191, 193, 312, 314
	**Pocket 2**	Chain A: 142, 145, 146, 147, 148, 149, 150, 151, 153, 154, 155, 156, 157, 168, 171, 172, 175, 176, 179, 180, 183<br>Chain B: 142, 146, 147, 149, 150, 151, 152, 153, 155, 156, 157, 168, 171, 172, 175, 176, 179, 97<br>Chain C: 108, 143, 146, 147, 148, 149, 151, 168, 171, 172, 173, 175, 176, 179<br>Chain D: 147, 148, 149, 150, 151

Table S4. Predicted binding affinities (kcal/mol) of the compounds screened against the central cavity region of the GIRK4^{WT} and GIRK4^{G151E} channels.

	GIRK4^{WT}	GIRK4^{G151E}	
**Compound**	**Binding Affinity (kcal/mol)**	**Binding Affinity (kcal/mol)**	**Classification**
Citrostadienol	-8.7	-8.8	**4-Methylsterols**
24-methyl-24(25)-dehydrolophenol	-8.8	-8.3	**4-Methylsterols**
28-isocitrostadienol	-9.1	-8.0	**4-Methylsterols**
24-Ethyl-E-23-dehydrolophenol	-8.9	-7.9	**4-Methylsterols**
24-methyl-(E)-23-dehydrolophenol	-8.8	-7.9	**4-Methylsterols**
24-Methylenelophenol	-8.2	-7.9	**4-Methylsterols**
Gramisterol	-7.7	-7.9	**4-Methylsterols**
24-methyl-31-nor-9(11)-lanostenol	-7.5	-7.9	**4-Methylsterols**
24-methylene-31-nor-9(11)-lanostenol	-7.2	-7.9	**4-Methylsterols**
24-Ethylidenelophenol	-8.1	-7.7	**4-Methylsterols**
24-ethyllophenol	-8.6	-7.6	**4-Methylsterols**
Obtusifoliol	-7.3	-7.6	**4-Methylsterols**
Cycloeucalenol	-7.0	-6.7	**4-Methylsterols**
Terpineol	-5.6	-5.4	**Alcohols**
Lavendulol	-5.5	-5.3	**Alcohols**
2-Phenylethanol	-5.4	-4.8	**Alcohols**
6-Methyl-5-hepten-3-ol	-5.2	-4.7	**Alcohols**
3-Methyl-1-butanol	-4.0	-4.7	**Alcohols**
Decanol	-5.2	-4.6	**Alcohols**
2-Octanol	-5.1	-4.6	**Alcohols**
3-Octenol	-5.2	-4.5	**Alcohols**
Nonanol	-5.2	-4.5	**Alcohols**
2-Methyl-1-butanol	-4.2	-4.5	**Alcohols**
Benzyl alcohol	-5.1	-4.4	**Alcohols**
2-Heptanol	-4.9	-4.4	**Alcohols**
Octanol	-4.7	-4.4	**Alcohols**
Heptanol	-4.6	-4.4	**Alcohols**
Heptan-2-ol	-4.9	-4.3	**Alcohols**
4-Hexenol	-4.5	-4.3	**Alcohols**
cis-2-Hexenol	-4.5	-4.3	**Alcohols**
trans-3-Hexenol	-4.4	-4.3	**Alcohols**
trans-2-Hexenol	-4.4	-4.2	**Alcohols**
cis-3-Hexenol	-4.3	-4.2	**Alcohols**
2-Penten-1-ol	-4.1	-4.2	**Alcohols**

Butan-1-ol	-3.7	-4.1	**Alcohols**
Hexanol	-4.2	-4.0	**Alcohols**
Pentanol	-4.0	-4.0	**Alcohols**
3-Pentanol	-4.4	-3.9	**Alcohols**
3-Penten-2-ol	-4.3	-3.9	**Alcohols**
2-Methyl-3-butenol	-4.2	-3.9	**Alcohols**
Butan-2-ol	-3.8	-3.9	**Alcohols**
1-Penten-3-ol	-4.4	-3.8	**Alcohols**
1-Propanol	-3.3	-3.4	**Alcohols**
Ethanol	-2.8	-2.9	**Alcohols**
Methanol	-2.2	-2.2	**Alcohols**
2,4 Hexadienal	-5.7	-5.1	**Aldehydes**
cis-2-Nonenal	-5.6	-5.1	**Aldehydes**
trans-4,5-Epoxy-trans-2-decenal	-6.3	-5.0	**Aldehydes**
2,4-Decadienal	-5.4	-4.9	**Aldehydes**
Vanillin	-5.7	-4.8	**Aldehydes**
trans-2-Decenal	-5.6	-4.8	**Aldehydes**
2,4-Heptadienal	-5.5	-4.8	**Aldehydes**
trans,trans-2,4-Decadienal	-5.5	-4.7	**Aldehydes**
trans-2-Nonenal	-5.4	-4.7	**Aldehydes**
Nonanal	-5.3	-4.7	**Aldehydes**
Benzaldehyde	-4.1	-4.7	**Aldehydes**
trans-2-Octenal	-5.1	-4.6	**Aldehydes**
Decanal	-5.3	-4.5	**Aldehydes**
Phenylacetaldehyde	-5.1	-4.5	**Aldehydes**
2-Methylbutanal	-5.0	-4.5	**Aldehydes**
2-Methyl-2-butenal	-4.6	-4.5	**Aldehydes**
trans-2-Heptenal	-4.9	-4.4	**Aldehydes**
Propanal	-4.8	-4.4	**Aldehydes**
cis-3-Hexenal	-4.6	-4.4	**Aldehydes**
Hexanal	-4.6	-4.4	**Aldehydes**
cis-2-Hexenal	-5.2	-4.3	**Aldehydes**
cis-2-Pentenal	-4.8	-4.3	**Aldehydes**
Octanal	-4.7	-4.3	**Aldehydes**
3-Methylbutanal	-4.1	-4.3	**Aldehydes**
trans-2-Hexenal	-4.5	-4.2	**Aldehydes**
Pentanal	-4.4	-4.2	**Aldehydes**
Acetaldehyde	-4.5	-4.0	**Aldehydes**
3-Hexanal	-4.2	-4.0	**Aldehydes**
Heptanal	-4.1	-4.0	**Aldehydes**
trans-2-Pentenal	-4.1	-4.0	**Aldehydes**
trans,trans-2,4-Nonadienal	-3.7	-4.0	**Aldehydes**
trans,cis-2,4-Decadienal	-4.0	-3.8	**Aldehydes**
cis-2-Heptenal	-2.6	-2.9	**Aldehydes**

Hexacosane	-4.9	-5.0	**Alkanes**
Tetradecane	-4.5	-4.7	**Alkanes**
Styrene	-5.2	-4.6	**Alkanes**
Tridecane	-4.7	-4.6	**Alkanes**
Dodecane	-5.2	-4.5	**Alkanes**
2-Methylbutane	-3.7	-4.5	**Alkanes**
Triacontane	-5.2	-4.4	**Alkanes**
Pentacosane	-5.0	-4.4	**Alkanes**
Tricosane	-5.0	-4.4	**Alkanes**
Pentadecane	-4.9	-4.4	**Alkanes**
Heptadecane	-4.8	-4.4	**Alkanes**
Octadecane	-5.3	-4.3	**Alkanes**
Tetratriacontane	-5.3	-4.3	**Alkanes**
Hentriacontane	-5.1	-4.3	**Alkanes**
Docosane	-5.0	-4.3	**Alkanes**
Dotriacontane	-5.0	-4.3	**Alkanes**
Nonacosane	-5.0	-4.3	**Alkanes**
Hexadecane	-4.9	-4.3	**Alkanes**
Heneicosane	-4.8	-4.3	**Alkanes**
2-Methylpentane	-4.2	-4.3	**Alkanes**
Heptacosane	-5.0	-4.2	**Alkanes**
Octacosane	-4.9	-4.2	**Alkanes**
Icosane	-5.2	-4.1	**Alkanes**
Tetracosane	-5.0	-4.1	**Alkanes**
Nonadecane	-4.9	-4.1	**Alkanes**
Nonane	-4.9	-4.1	**Alkanes**
Methyl benzene	-4.8	-4.1	**Alkanes**
Octane	-4.4	-4.0	**Alkanes**
Tritriacontane	-4.9	-3.9	**Alkanes**
Heptane	-4.4	-3.8	**Alkanes**
3-Methylpentane	-4.1	-3.7	**Alkanes**
Hexane	-4.1	-3.7	**Alkanes**
Hexene	-4.1	-3.7	**Alkanes**
6,10-dimethyl-1-undecene	-5.1	-5.0	**Alkenes**
9-Hexacosene	-5.2	-4.5	**Alkenes**
9-Pentacosene	-5.1	-4.4	**Alkenes**
9-Tricosene	-5.0	-4.3	**Alkenes**
9-Docosene	-5.3	-4.2	**Alkenes**
9-Heptacosene	-5.4	-4.1	**Alkenes**
9-Tetracosene	-5.1	-4.1	**Alkenes**
8-Heptadecene	-5.2	-4.0	**Alkenes**
Tyrosine	-6.2	-6.2	**Amino Acids**
Arginine	-6.2	-6.1	**Amino Acids**
Phenylalanine	-6.2	-5.8	**Amino Acids**

Glutamine	-5.4	-5.4	**Amino Acids**
Glutamic acid	-5.5	-5.2	**Amino Acids**
Aspargine	-5.2	-4.9	**Amino Acids**
Valine	-4.7	-4.9	**Amino Acids**
Leucine	-5.1	-4.8	**Amino Acids**
Aspartic acid	-4.9	-4.8	**Amino Acids**
Isoleucine	-5.0	-4.7	**Amino Acids**
Serine	-4.7	-4.4	**Amino Acids**
Alanine	-4.5	-4.4	**Amino Acids**
Piperaquine	-7.6	-9.6	**Antibiotics**
Cefditoren Pivoxil	-8.6	-9.0	**Antibiotics**
Fusidic acid	-7.7	-9.0	**Antibiotics**
Ceftriaxone	-8.2	-8.6	**Antibiotics**
Pyronaridine	-9.3	-8.5	**Antibiotics**
Polymyxin B1	-9.2	-8.4	**Antibiotics**
Atovaquone	-8.4	-8.4	**Antibiotics**
Mefloquine	-7.7	-8.3	**Antibiotics**
Doxycycline	-8.6	-8.2	**Antibiotics**
Moxifloxacin	-7.5	-8.2	**Antibiotics**
Biltricide	-8.4	-8.1	**Antibiotics**
Cefuroxime	-7.3	-8.0	**Antibiotics**
Cefamandole	-7.5	-7.9	**Antibiotics**
Gemifloxacin	-7.4	-7.9	**Antibiotics**
Ofloxacin	-8.0	-7.8	**Antibiotics**
Cefotaxime	-7.5	-7.8	**Antibiotics**
Levofloxacin	-7.4	-7.8	**Antibiotics**
Gatifloxacin	-7.0	-7.8	**Antibiotics**
Ciprofloxacin	-7.5	-7.7	**Antibiotics**
Artemisinin	-7.3	-7.7	**Antibiotics**
Ampicillin	-7.6	-7.6	**Antibiotics**
Cefaclor	-7.4	-7.6	**Antibiotics**
Norfloxacin	-7.4	-7.6	**Antibiotics**
Quinine	-7.8	-7.4	**Antibiotics**
Linezolid	-7.5	-7.4	**Antibiotics**
Amodiaquine	-7.6	-7.2	**Antibiotics**
Benzylpenicillin	-7.4	-7.2	**Antibiotics**
Tafenoquine	-8.4	-7.1	**Antibiotics**
Amoxicillin	-8.0	-7.1	**Antibiotics**
Sulfamethoxazole	-7.0	-7.1	**Antibiotics**
Oxolinic acid	-6.9	-7.1	**Antibiotics**
Quinacrine	-7.3	-7.0	**Antibiotics**
Niclosamide	-8.2	-6.9	**Antibiotics**
Nitazoxanide	-8.0	-6.9	**Antibiotics**
Tizoxanide	-7.6	-6.9	**Antibiotics**

Lumefantrine	-7.3	-6.9	**Antibiotics**
Trimethoprim	-7.1	-6.8	**Antibiotics**
Pyrimethamine	-7.0	-6.8	**Antibiotics**
Chloramphenicol	-6.9	-6.6	**Antibiotics**
Proguanil	-6.5	-6.5	**Antibiotics**
Hydroxychloroquine	-6.8	-6.4	**Antibiotics**
Terbinafine	-6.9	-6.0	**Antibiotics**
Chloroquine	-6.8	-6.0	**Antibiotics**
Levamisole	-6.1	-5.8	**Antibiotics**
Clioquinol	-5.9	-5.6	**Antibiotics**
Metronidazole	-5.9	-5.2	**Antibiotics**
Pyrazinamide	-5.3	-4.7	**Antibiotics**
3-Methyl-butanoic acid	-4.5	-5.1	**Carboxylic Acids**
Octanoic acid	-5.3	-5.0	**Carboxylic Acids**
3-Methylbutyric acid	-4.5	-5.0	**Carboxylic Acids**
Heptanoic acid	-5.2	-4.9	**Carboxylic Acids**
Hexanoic acid	-4.9	-4.9	**Carboxylic Acids**
Pentanoic acid	-4.6	-4.5	**Carboxylic Acids**
Isobutyric acid	-4.3	-4.5	**Carboxylic Acids**
Butanoic acid	-4.2	-4.4	**Carboxylic Acids**
Propanoic acid	-3.8	-3.8	**Carboxylic Acids**
Acetic acid	-3.4	-3.6	**Carboxylic Acids**
Lutein	-5.8	-8.1	**Carotenoids**
β-Carotene	-5.8	-7.8	**Carotenoids**
Chlorophyllide a	-8.8	-8.9	**Chlorophylls**
Chlorophyllide b	-8.7	-8.9	**Chlorophylls**
Pheophorbide b	-8.7	-8.7	**Chlorophylls**
Chlorophyll a	-8.4	-8.6	**Chlorophylls**
Chlorophyll b	-8.5	-8.4	**Chlorophylls**
Pheophorbide a	-8.5	-8.4	**Chlorophylls**
Pyropheophytin α	-8.1	-8.4	**Chlorophylls**
Pheophytin b	-7.7	-8.0	**Chlorophylls**
Pheophytin α	-7.2	-7.6	**Chlorophylls**
24-Methylene-cholesterol	-8.6	-9.0	**Common Sterols**
Ergosterol	-8.5	-8.2	**Common Sterols**
Δ-5,23-Stigmastadienol	-8.0	-8.2	**Common Sterols**
β-sitosterol	-8.6	-8.1	**Common Sterols**
Stigmastanol	-8.4	-8.0	**Common Sterols**
Δ7,22-Ergostadienol	-8.6	-7.9	**Common Sterols**
Δ-5-Avenasterol	-8.0	-7.9	**Common Sterols**
Clerosterol	-7.2	-7.9	**Common Sterols**
Δ-5,24-Stigmastadienol	-7.8	-7.8	**Common Sterols**
22,23-Dihydrobrassicasterol	-7.7	-7.8	**Common Sterols**
Campestanol	-7.6	-7.8	**Common Sterols**

Δ-7-Avenasterol	-8.8	-7.6	**Common Sterols**
Campesterol	-7.5	-7.6	**Common Sterols**
Δ-7-Stigmastenol	-8.4	-7.5	**Common Sterols**
Δ7,24-Ergostadienol	-8.5	-7.4	**Common Sterols**
Brassicasterol	-7.7	-7.4	**Common Sterols**
Cholesterol	-7.2	-7.3	**Common Sterols**
Stigmasterol	-7.1	-7.1	**Common Sterols**
Scopolin	-7.6	-7.9	**Coumarins**
Esculin	-7.3	-7.7	**Coumarins**
Scopoletin	-7.2	-6.3	**Coumarins**
Esculetin	-7.3	-6.0	**Coumarins**
1,3-diacylglycerol	-5.4	-5.0	**Diacylglycerols**
2,3-diacylglycerol	-5.4	-5.0	**Diacylglycerols**
1,2-diacylglycerol	-5.4	-4.8	**Diacylglycerols**
D-(+)-lactose	-6.7	-7.2	**Diasaccharides**
D-(+)-sucrose	-6.7	-7.0	**Diasaccharides**
Geranylgeraniol	-6.4	-5.9	**Diterpene Alcohols**
Phytol	-5.9	-5.2	**Diterpene Alcohols**
Ethyl cinnamate	-6.6	-5.5	**Esters**
2-Ethylphenyl acetate	-6.3	-5.3	**Esters**
Benzyl acetate	-6.0	-5.3	**Esters**
Phenethyl acetate	-5.9	-5.3	**Esters**
cis-3-Hexenyl acetate	-6.3	-5.1	**Esters**
Ethyl cyclohexylcarboxylate	-5.4	-5.1	**Esters**
Ethyl octanoate	-4.9	-4.9	**Esters**
2-Hexenyl acetate	-5.3	-4.8	**Esters**
3-Hexenyl acetate	-5.3	-4.8	**Esters**
Isopentyl acetate	-5.1	-4.7	**Esters**
2-Methylpropyl butanoate	-5.3	-4.6	**Esters**
Methyl octanoate	-5.3	-4.6	**Esters**
Octyl acetate	-5.2	-4.6	**Esters**
Hexyl acetate	-5.0	-4.6	**Esters**
Ethyl hexanoate	-5.4	-4.5	**Esters**
Methyl heptanoate	-5.3	-4.4	**Esters**
2-Methylbutyl acetate	-5.1	-4.4	**Esters**
Propyl 2-methylpropanoate	-4.7	-4.4	**Esters**
Propyl butanoate	-5.1	-4.3	**Esters**
Ethyl 3-methylbutanoate	-4.8	-4.3	**Esters**
Butyl acetate	-4.7	-4.3	**Esters**
Methyl hexanoate	-5.0	-4.2	**Esters**
Ethyl 2-methylbutanoate	-4.9	-4.2	**Esters**
Ethyl isobutyrate	-4.6	-4.2	**Esters**
Methyl 3-methylbutanoate	-4.6	-4.2	**Esters**
Propyl propanoate	-4.8	-4.1	**Esters**

Methyl pentanoate	-4.7	-4.1	**Esters**
Ethyl butanoate	-4.9	-4.0	**Esters**
Ethyl-2-methypropanoate	-4.6	-4.0	**Esters**
Ethyl propanoate	-4.5	-4.0	**Esters**
Methyl butanoate	-4.5	-4.0	**Esters**
Methyl 2-methylbutanoate	-4.7	-3.9	**Esters**
Ethyl acetate	-4.1	-3.8	**Esters**
Methyl acetate	-3.6	-3.7	**Esters**
1,8-Cineole	-4.8	-5.3	**Ethers**
Diethyl ether	-3.4	-2.9	**Ethers**
Ethyl palmitate	-5.5	-4.5	**Ethyl Esters**
Ethyl linoleate	-5.4	-4.5	**Ethyl Esters**
Ethyl oleate	-5.7	-4.4	**Ethyl Esters**
Ethyl stearate	-5.2	-4.4	**Ethyl Esters**
Octacosanol	-5.0	-5.0	**Fatty Alcohols**
Pentacosanol	-5.8	-4.6	**Fatty Alcohols**
Tricosanol	-5.3	-4.6	**Fatty Alcohols**
Heptacosanol	-5.1	-4.6	**Fatty Alcohols**
Eicosanol	-5.4	-4.4	**Fatty Alcohols**
Docosanol	-5.2	-4.4	**Fatty Alcohols**
Hexacosanol	-5.0	-4.4	**Fatty Alcohols**
Tetracosanol	-5.2	-4.3	**Fatty Alcohols**
Rutin	-9.0	-10.2	**Flavonoids**
Hesperidin	-10.2	-10.1	**Flavonoids**
Diosmin	-10.3	-10.0	**Flavonoids**
Apigenin-7-O-rutinoside	-10.1	-10.0	**Flavonoids**
Luteolin-4’-O-glucoside	-10.1	-9.8	**Flavonoids**
Luteolin-7,4-O-diglucoside	-9.5	-9.8	**Flavonoids**
Scolymoside	-10.0	-9.6	**Flavonoids**
Luteolin-7-O-rutinoside	-10.7	-9.5	**Flavonoids**
Luteolin-3′,7-O-diglucoside	-9.6	-9.5	**Flavonoids**
Hidrosmin	-9.7	-9.4	**Flavonoids**
Silibinin	-8.8	-9.3	**Flavonoids**
Icariin	-8.6	-9.3	**Flavonoids**
Quercetin 3-O-rutinoside	-9.2	-9.0	**Flavonoids**
Monoxerutin	-9.0	-9.0	**Flavonoids**
Cyanidin-3-O-rutinoside	-9.4	-8.9	**Flavonoids**
Luteolin-7-O-glucoside	-9.0	-8.9	**Flavonoids**
Luteolin-4'-O-rutinoside	-8.7	-8.9	**Flavonoids**
Apigenin trimethylether	-8.8	-8.7	**Flavonoids**
Luteolin-8-C-glucoside	-8.7	-8.7	**Flavonoids**
Vicenin-2	-9.2	-8.6	**Flavonoids**
Luteolin-6-C-glucoside	-8.8	-8.6	**Flavonoids**
Epigallocatechin gallate	-8.7	-8.6	**Flavonoids**
Chrysoeriol-7-O-glucoside	-8.6	-8.6	**Flavonoids**

Quercetin-3-O-glucoside	-8.1	-8.6	**Flavonoids**
Quercitrin	-8.1	-8.6	**Flavonoids**
Cyanidin-3-O-glucoside	-8.4	-8.5	**Flavonoids**
Isoquercetin	-8.1	-8.5	**Flavonoids**
Quercetin-3'-O-phosphate	-8.1	-8.4	**Flavonoids**
Daidzin	-8.6	-8.3	**Flavonoids**
Myricetin	-8.1	-8.3	**Flavonoids**
Delphinidin-3-O-glucoside	-7.6	-8.3	**Flavonoids**
Troxerutin	-8.6	-8.2	**Flavonoids**
Sofalcone	-8.4	-8.2	**Flavonoids**
Isorhamnetin	-7.7	-8.2	**Flavonoids**
alpha-Naphthoflavone	-9.0	-8.1	**Flavonoids**
Eriodictyol	-8.0	-8.1	**Flavonoids**
Morin	-7.9	-8.1	**Flavonoids**
Quercetin	-7.9	-8.1	**Flavonoids**
Crofelemer	-8.0	-8.0	**Flavonoids**
Luteolin	-8.0	-8.0	**Flavonoids**
Taxifolin	-7.8	-8.0	**Flavonoids**
Recoflavone	-7.7	-8.0	**Flavonoids**
Chrysoeriol	-8.2	-7.9	**Flavonoids**
Acacetin diacetate	-8.1	-7.9	**Flavonoids**
Rhamnetin	-8.0	-7.9	**Flavonoids**
Tricetin	-8.0	-7.9	**Flavonoids**
Methoxyluteolin	-7.9	-7.9	**Flavonoids**
Kaempherol	-7.8	-7.9	**Flavonoids**
beta-Naphthoflavone	-8.5	-7.8	**Flavonoids**
Dihydromyricetin	-7.9	-7.8	**Flavonoids**
7-(alpha-D-Glucopyranosyloxy)-4-methyl-2H-1-benzopyran-2-one	-7.7	-7.8	**Flavonoids**
4-methylumbelliferyl beta-D-glucoside	-7.4	-7.8	**Flavonoids**
Icaritin	-8.4	-7.7	**Flavonoids**
Apigenin	-7.9	-7.7	**Flavonoids**
Hispidulin	-7.9	-7.7	**Flavonoids**
Delphinidin	-7.8	-7.7	**Flavonoids**
Genkwanin	-7.8	-7.7	**Flavonoids**
Cianidanol	-7.5	-7.7	**Flavonoids**
ME-344	-7.2	-7.7	**Flavonoids**
Diosmetin	-8.0	-7.6	**Flavonoids**
Cyanidin (cation)	-7.7	-7.6	**Flavonoids**
Epigallocatechin	-7.5	-7.6	**Flavonoids**
Baicalein	-7.4	-7.6	**Flavonoids**
Flavoxate	-8.2	-7.5	**Flavonoids**

5,7,2'-trihydroxy-6,8-dimethoxyflavone	-7.8	-7.5	**Flavonoids**
Naringenin	-7.8	-7.5	**Flavonoids**
Sakuranetin	-7.8	-7.5	**Flavonoids**
Chrysin	-7.7	-7.5	**Flavonoids**
Fisetin	-7.7	-7.5	**Flavonoids**
Hesperetin	-7.6	-7.5	**Flavonoids**
Acacetin	-7.5	-7.5	**Flavonoids**
5-deoxyflavanone	-7.8	-7.4	**Flavonoids**
Hesperitin	-7.6	-7.4	**Flavonoids**
Elafibranor	-7.2	-7.4	**Flavonoids**
Epicatechin	-7.9	-7.3	**Flavonoids**
Phloretin	-7.9	-7.3	**Flavonoids**
(2S)-7-hydroxyflavanone	-7.7	-7.3	**Flavonoids**
Idronoxil	-7.7	-7.3	**Flavonoids**
Biochanin A	-7.5	-7.3	**Flavonoids**
Triphendiol	-7.4	-7.3	**Flavonoids**
Dihydroxymethoxychalcone	-7.3	-7.3	**Flavonoids**
Eupatilin	-7.3	-7.3	**Flavonoids**
Efloxate	-7.2	-7.3	**Flavonoids**
Flavone	-7.8	-7.2	**Flavonoids**
Equol	-7.7	-7.2	**Flavonoids**
Lucidumoside C	-7.1	-7.2	**Flavonoids**
4'-Hydroxyflavanone	-7.9	-7.1	**Flavonoids**
PD-98059	-7.7	-7.1	**Flavonoids**
Apigenin-7-O-glucoside	-7.5	-7.1	**Flavonoids**
Isoformononetin	-7.9	-6.9	**Flavonoids**
Genistein	-7.8	-6.8	**Flavonoids**
Isoflavone	-7.4	-6.8	**Flavonoids**
Ipriflavone	-7.7	-6.7	**Flavonoids**
Formononetin	-7.2	-6.7	**Flavonoids**
3,4-methyl-3-pentenyl furan	-5.9	-5.6	**Furans**
3-Methyl-2-pentylfuran	-5.6	-4.9	**Furans**
3-Propylfuran	-4.9	-4.7	**Furans**
2-Propylfuran	-4.9	-4.4	**Furans**
2-Ethylfuran	-4.6	-4.2	**Furans**
Oxidized isoverbascoside	-9.2	-10.0	**Glucosides**
Isoacteoside	-9.5	-9.5	**Glucosides**
4'-O-β-D-Glucosyl-9-O-(6''-deoxysaccharosyl)olivil	-9.3	-9.5	**Glucosides**
Oxidized verbacoside	-9.6	-9.2	**Glucosides**
Actoside	-9.1	-9.2	**Glucosides**
Isoverbascoside	-9.6	-9.1	**Glucosides**

Verbascoside	-9.1	-9.1	**Glucosides**
Quercetin-3-rhamnoside	-8.4	-9.1	**Glucosides**
Orbanchoside	-9.6	-8.9	**Glucosides**
Hellicoside	-9.2	-8.9	**Glucosides**
6'-β-D-Glucopyranosyl oleoside	-8.1	-8.9	**Glucosides**
Suspensaside	-9.4	-8.6	**Glucosides**
6'-O-[(2E)-2,6-Dimethyl-8-hydroxy- 2-octenoyloxy]-secologanoside	-7.5	-8.5	**Glucosides**
Quercetin-7-O-glucoside	-9.1	-8.4	**Glucosides**
Caffeoyl-6'-secologanoside	-8.7	-8.2	**Glucosides**
Comselogoside	-8.5	-8.1	**Glucosides**
Wedelosin	-10.1	-7.9	**Glucosides**
Hydroxytyrosol diglucoside	-8.6	-7.7	**Glucosides**
Oleoside-11-Methylester	-7.4	-7.7	**Glucosides**
6'-Rhamnopyranosyl oleoside	-7.5	-7.6	**Glucosides**
Oleoside	-7.5	-7.6	**Glucosides**
Secologanoside	-7.2	-7.4	**Glucosides**
Verucosin	-7.5	-7.3	**Glucosides**
Hydroxytyrosol-3-β-glucoside	-7.0	-7.3	**Glucosides**
Hydroxytyrosol rhamnoside	-7.6	-7.2	**Glucosides**
Hydroxytyrosol-1′-β-glucoside	-7.6	-7.2	**Glucosides**
Hydroxytyrosol-4-β-glucoside	-7.0	-7.1	**Glucosides**
Salidroside	-7.4	-6.8	**Glucosides**
2,3-dihydrocaffeic acid	-6.3	-6.1	**Hydroxybenzoic Acids**
2,4 dihydroxybenzoic acid	-6.3	-5.9	**Hydroxybenzoic Acids**
2,6-Dihydroxybenzoic acid	-6.0	-5.9	**Hydroxybenzoic Acids**
Gallic acid	-6.0	-5.9	**Hydroxybenzoic Acids**
Quinic acid	-5.4	-5.9	**Hydroxybenzoic Acids**
Phloretic acid	-6.0	-5.8	**Hydroxybenzoic Acids**
Shikimic acid	-5.5	-5.8	**Hydroxybenzoic Acids**
Gentisic acid	-6.2	-5.7	**Hydroxybenzoic Acids**
4-O-methyl-D-glucuronic acid	-6.1	-5.7	**Hydroxybenzoic Acids**

Protocatechuic acid	-6.2	-5.5	**Hydroxybenzoic Acids**
Vanillic acid	-6.1	-5.4	**Hydroxybenzoic Acids**
Syringic acid	-5.7	-5.4	**Hydroxybenzoic Acids**
4-hydroxybenzoic acid	-6.0	-5.3	**Hydroxybenzoic Acids**
β-Hydroxy verbascoside	-8.8	-9.2	**Hydroxycinnamic Acids**
Chlorogenic acid	-7.8	-7.8	**Hydroxycinnamic Acids**
Caffeoylglucose	-7.9	-7.7	**Hydroxycinnamic Acids**
Rosmarinic acid	-7.8	-7.6	**Hydroxycinnamic Acids**
Caftaric acid	-7.7	-7.3	**Hydroxycinnamic Acids**
m-Coumaric acid	-6.8	-6.7	**Hydroxycinnamic Acids**
Caffeic acid	-6.5	-6.7	**Hydroxycinnamic Acids**
Hydroxycaffeic acid	-6.8	-6.6	**Hydroxycinnamic Acids**
Ferulic acid	-6.4	-6.2	**Hydroxycinnamic Acids**
Sinapic acid	-5.8	-6.1	**Hydroxycinnamic Acids**
o-Coumaric acid	-6.5	-6.0	**Hydroxycinnamic Acids**
p-Coumaric acid	-6.3	-6.0	**Hydroxycinnamic Acids**
Cinnamic acid	-6.5	-5.9	**Hydroxycinnamic Acids**
Dihydro-p-coumaric acid	-6.1	-5.7	**Hydroxycinnamic Acids**
1-(3’-Methoxy-4’-hydroxy)- phenyl-6,7-dihydroxyisochroman	-7.1	-6.8	**Hydroxy-Isochromans**
1-Phenyl-6,7-dihydroxyisochroman	-6.9	-6.8	**Hydroxy-Isochromans**
3,4-Dihydroxyphenylacetic acid	-5.9	-5.8	**Hydroxyphenylacetic Acids**
Homovanillic acid	-5.6	-5.8	**Hydroxyphenylacetic Acids**
4-Hydroxy-3-methoxy-phenylacetic acid	-6.0	-5.7	**Hydroxyphenylacetic Acids**
p-Hydroxyphenylacetic acid	-5.6	-5.5	**Hydroxyphenylacetic Acids**

Homoveratric acid	-6.3	-5.4	**Hydroxyphenylacetic Acids**
2,5-Dihydroxyphenylacetic acid	-5.9	-5.3	**Hydroxyphenylacetic Acids**
Loganic acid	-7.3	-8.0	**Iridoids**
Loganin	-7.5	-7.3	**Iridoids**
trans-β-Damascenone	-5.8	-5.6	**Ketones**
6-Methyl-5-hepten-2-one	-5.3	-5.1	**Ketones**
Acetophenone	-5.6	-4.7	**Ketones**
cis-1,5-Octadien-3-one	-5.4	-4.7	**Ketones**
Octan-2-one	-4.9	-4.6	**Ketones**
1-Octen-3-one	-5.2	-4.5	**Ketones**
2-Nonanone	-5.1	-4.5	**Ketones**
2-Octanone	-5.0	-4.5	**Ketones**
Heptan-2-one	-4.7	-4.5	**Ketones**
4-Methyl-2-pentanone	-4.5	-4.5	**Ketones**
3-Octanone	-5.1	-4.3	**Ketones**
2-Heptanone	-4.6	-4.3	**Ketones**
2-Hexanone	-4.6	-4.3	**Ketones**
3-Methyl-2-butanone	-4.1	-4.3	**Ketones**
2-Butanone	-3.7	-4.0	**Ketones**
Butan-2-one	-3.7	-4.0	**Ketones**
1-Penten-3-one	-4.3	-3.8	**Ketones**
3-Pentanone	-4.2	-3.7	**Ketones**
(+)-1-Hydroxypinoresinol-4''-O-methyl ether	-7.9	-8.7	**Lignans**
(+)-1-Acetoxypinoresinol-4'-β-D-glucopyranoside	-7.9	-8.6	**Lignans**
(+)-1-Acetoxypinoresinol-4'-β-D-glucopyranoside-4''-O-methyl ether	-7.9	-8.6	**Lignans**
(+)-1-Hydroxypinoresinol-4'-β-D-glucopyranoside	-8.4	-8.4	**Lignans**
(+)-Fraxiresinol-1-β-D-glucopyranoside	-8.4	-8.3	**Lignans**
Berchemol	-7.5	-8.0	**Lignans**
Syringaresinol	-7.4	-8.0	**Lignans**
1-Acetoxypinoresinol	-8.3	-7.8	**Lignans**
Pinoresinol	-7.8	-7.8	**Lignans**
(+)-1-Acetoxypinoresinol-4''-O-methyl ether	-7.7	-7.7	**Lignans**
3-Acetyloxy berchemol	-7.6	-7.4	**Lignans**
D-(+)-Erythro-1-(4-hydroxy-3-methoxy)- 214 -phenyl-1,2,3-propantriol	-7.3	-7.4	**Lignans**
(-)-Olivil	-7.3	-7.0	**Lignans**

Hydroxypinoresinol	-6.3	-6.3	**Lignans**
Linoleic	-6.0	-5.4	**Long Chain Fatty Acids**
11-*cis*-vaccenic	-5.5	-5.4	**Long Chain Fatty Acids**
Elaidic	-5.5	-5.4	**Long Chain Fatty Acids**
Myristic	-5.4	-5.4	**Long Chain Fatty Acids**
Gadoleic	-5.6	-5.3	**Long Chain Fatty Acids**
Palmitoleic	-5.6	-5.3	**Long Chain Fatty Acids**
Trans-palmitoleic	-5.6	-5.3	**Long Chain Fatty Acids**
Arachidic	-5.3	-5.3	**Long Chain Fatty Acids**
Stearic	-5.8	-5.2	**Long Chain Fatty Acids**
Linolenic	-6.0	-5.0	**Long Chain Fatty Acids**
Linoelaidic	-5.9	-5.0	**Long Chain Fatty Acids**
Eicosenoic	-5.7	-5.0	**Long Chain Fatty Acids**
Petroselinic	-5.5	-5.0	**Long Chain Fatty Acids**
Palmitic	-5.8	-4.9	**Long Chain Fatty Acids**
Oleic	-5.7	-4.7	**Long Chain Fatty Acids**
Margaric acid	-5.6	-4.7	**Long Chain Fatty Acids**
Cis-Heptadecenoic	-5.6	-4.6	**Long Chain Fatty Acids**
Behenic	-5.2	-4.6	**Long Chain Fatty Acids**
Erucic	-5.5	-4.5	**Long Chain Fatty Acids**
Lignoceric	-5.3	-4.4	**Long Chain Fatty Acids**
Troleandomycin	-8.5	-10.1	**Macrolides**
Ivermectin	-9.6	-10.0	**Macrolides**
Telithromycin	-9.2	-9.6	**Macrolides**
Erythromycin	-8.2	-9.6	**Macrolides**
Clarithromycin	-8.2	-9.4	**Macrolides**
Idremcinal	-8.6	-9.2	**Macrolides**

Pseudo erythromycin A enol ether	-8.8	-8.8	**Macrolides**
Anhydroerythromycin A	-8.7	-8.7	**Macrolides**
Erythromycin B	-8.5	-8.7	**Macrolides**
Flurithromycin	-8.9	-8.6	**Macrolides**
Josamycin	-8.6	-8.6	**Macrolides**
Azithromycin	-8.2	-8.5	**Macrolides**
Erythromycin A oxime	-9.1	-8.4	**Macrolides**
Erythromycin C	-8.9	-8.3	**Macrolides**
Roxithromycin D7	-8.9	-8.2	**Macrolides**
Decladinose roxithromycin	-8.1	-8.1	**Macrolides**
N-demethyl roxithromycin	-7.9	-8.1	**Macrolides**
Roxithromycin	-8.4	-7.9	**Macrolides**
Dirithromycin	-8.3	-7.4	**Macrolides**
Clindamycin	-6.9	-7.0	**Macrolides**
Rapamycin	-10.4	-6.1	**Macrolides**
Lauric	-5.6	-4.9	**Medium Chain Fatty Acids**
Isoeugenol	-6.0	-5.5	**Methoxyphenols**
Homovanillin	-5.7	-5.2	**Methoxyphenols**
2-Methoxy-4-vinylphenol	-5.6	-4.9	**Methoxyphenols**
Guaiacol	-5.1	-4.7	**Methoxyphenols**
Methyl stearate	-5.0	-4.9	**Methyl Esters**
Methyl oleate	-5.3	-4.7	**Methyl Esters**
Methyl heptadecanoate	-5.6	-4.4	**Methyl Esters**
Methyl linoleate	-5.4	-4.4	**Methyl Esters**
Methyl palmitate	-5.2	-4.4	**Methyl Esters**
3-monoacylglycerol	-4.3	-5.0	**Monocylglycerols**
1-monoacylglycerol	-4.3	-4.6	**Monocylglycerols**
2-monoacylglycerol	-3.6	-4.4	**Monocylglycerols**
D-(-)-fructose	-5.5	-5.7	**Monosaccharides**
Sedoheptulose	-5.8	-5.6	**Monosaccharides**
D-(+)-glucose	-6.1	-5.5	**Monosaccharides**
D-(+)-mannose	-6.1	-5.4	**Monosaccharides**
D-(-)-galactose	-5.6	-5.3	**Monosaccharides**
1,6-anhydro-β-D-glucose	-5.4	-5.3	**Monosaccharides**
D-(+)-xylose	-5.1	-5.3	**Monosaccharides**
D-(-)-arabinose	-5.5	-5.2	**Monosaccharides**
Mannan	-8.3	-8.4	**Oligosaccharides**
α-Cellulose	-7.2	-7.2	**Oligosaccharides**
Galacturonan	-6.0	-6.2	**Oligosaccharides**
Pectin	-5.4	-5.8	**Oligosaccharides**
Citric acid	-6.0	-5.5	**Organic Acids**
Gluconic acid	-5.8	-5.4	**Organic Acids**
Succinic acid	-5.1	-4.6	**Organic Acids**

Oxalic acid	-4.4	-4.3	**Organic Acids**
Malic acid	-5.6	5.0	**Organic Acids**
Poly-unsaturated di-galactoside glycerol diester	-7.1	-7.0	**Other**
3-[1-(hydroxymethyl)-(E)-1-propenyl] glutaric acid	-5.9	-5.8	**Other**
Halleridone	-6.1	-5.3	**Other**
3-[1-(formyl)-(E)-1-propenyl] glutaric acid	-5.7	-5.2	**Other**
3-(1-Hydroxymethyl-1-propenyl)pentanedioic acid	-5.5	-5.2	**Other**
1,5-anhydroxylitol	-5.4	-4.8	**Other**
Deoxyloganic acid lauryl ester	-6.2	-5.5	**Phenolic Fatty Acid Esters**
1-oleyltyrosol	-6.5	-5.2	**Phenolic Fatty Acid Esters**
Phosphatidylinositol	-6.2	-6.3	**Phospholipids**
Lysophosphatidylethanolamine	-6.3	-5.9	**Phospholipids**
Phosphatidylcholine	-5.2	-5.6	**Phospholipids**
Phosphatidylglycerol	-5.4	-5.4	**Phospholipids**
Phosphatidic acid	-5.2	-5.2	**Phospholipids**
Phosphatidylethanolamine	-5.2	-5.2	**Phospholipids**
Lysophosphatidic acid	-5.6	-5.1	**Phospholipids**
Nüzhenide oleoside	-8.7	-9.8	**Secoiridoids**
Jaspolyoside	-9.2	-9.7	**Secoiridoids**
Isojaspolyoside A	-9.0	-9.7	**Secoiridoids**
Oleuropein diglucoside	-8.5	-9.6	**Secoiridoids**
Ligstroside derivative 3	-8.7	-9.5	**Secoiridoids**
Ligstroside derivative 4	-9.1	-9.2	**Secoiridoids**
Nüzhenide 11-Methyl oleoside	-8.9	-9.2	**Secoiridoids**
Ligstroside derivative 5	-8.2	-8.8	**Secoiridoids**
Elenolic acid diglucoside	-7.8	-8.7	**Secoiridoids**
Oleuropein dimer	-8.7	-8.6	**Secoiridoids**
Oleuricine A	-8.1	-8.6	**Secoiridoids**
Neo-nüzhenide	-9.3	-8.5	**Secoiridoids**
Nüzhenide	-8.8	-8.5	**Secoiridoids**
Ligstroside	-8.9	-8.4	**Secoiridoids**
Ligstroside-3'-O-β-D-glucopyranoside	-8.7	-8.4	**Secoiridoids**
Demethyloleuropein	-8.8	-8.3	**Secoiridoids**
Oleuropein	-8.9	-8.2	**Secoiridoids**
Jaspolyanoside	-9.9	-8.1	**Secoiridoids**
Oleuricine B	-9.0	-8.1	**Secoiridoids**
10-Hydroxyoleuropein	-8.2	-8.1	**Secoiridoids**

Oleuroside-10-carboxylic acid	-7.3	-8.1	**Secoiridoids**
7-Deoxyloganic acid	-7.2	-8.1	**Secoiridoids**
Demethylligstroside	-7.5	-8.0	**Secoiridoids**
Oleuropein-3''-Methyl ether	-7.9	-7.9	**Secoiridoids**
Oleuroside	-8.3	-7.8	**Secoiridoids**
7''-S-Hydroxyoleuropein	-9.0	-7.7	**Secoiridoids**
Dihydro-oleuropein	-8.1	-7.6	**Secoiridoids**
Ligstroside derivative 1	-8.6	-7.5	**Secoiridoids**
Demethyloleuropein aglycone	-7.7	-7.5	**Secoiridoids**
Elenolic acid glucoside	-7.2	-7.4	**Secoiridoids**
Oleoside dimethylester	-7.2	-7.4	**Secoiridoids**
Hydroxytyrosil elenolate	-7.9	-7.3	**Secoiridoids**
Oleuropein aglycone (3,4-DHPEA-EA)	-7.8	-7.2	**Secoiridoids**
10-Hydroxy oleuropein aglycone	-7.6	-7.2	**Secoiridoids**
10-Hydroxy oleuropein aglycone decarboxymethyl	-6.7	-7.2	**Secoiridoids**
Secologanic acid	-6.4	-7.2	**Secoiridoids**
3,4-DHPEA-DETA	-7.9	-7.1	**Secoiridoids**
10-Hydroxy-10-methyl oleuropein aglycone	-7.8	-7.1	**Secoiridoids**
Oleuropeindial Lactone (Cannizzaro-like product)	-7.8	-7.1	**Secoiridoids**
Oleuropein-3'-O-β-D-glucopyranoside	-8.8	-7.0	**Secoiridoids**
Ligstroside aglycone methyl acetal	-7.5	-7.0	**Secoiridoids**
Cornoside	-7.3	-7.0	**Secoiridoids**
Ligstroside aglycone	-7.0	-7.0	**Secoiridoids**
Ligstroside derivative 2	-6.8	-7.0	**Secoiridoids**
Monoaldehydic form of Oleuropein aglycon	-7.3	-6.9	**Secoiridoids**
Secologanol	-6.8	-6.9	**Secoiridoids**
Secologanin	-6.5	-6.8	**Secoiridoids**
Oleuropeindial (Cannizzaro-like product of oleuropeindial)	-7.2	-6.7	**Secoiridoids**
Monoaldehydic form of Ligstroside aglycon	-7.5	-6.6	**Secoiridoids**
demethyloleuropein aglycone (enol form)	-7.6	-6.5	**Secoiridoids**
Demethyloleuropein aglycone dialdehyde	-7.1	-6.5	**Secoiridoids**
Oleuropeindial (keto form)	-7.2	-6.4	**Secoiridoids**

Oleuropeindial (enol form)	-6.9	-6.4	**Secoiridoids**
Hemiacetal of dialdehydic oleuropein aglycone decarboxymethyl	-7.3	-6.3	**Secoiridoids**
Oleacein (Dialdehydic form of decarboxymethyl Oleuropein aglycon)	-7.1	-6.3	**Secoiridoids**
Methyl malate-hydroxytyrosol ester	-6.7	-6.3	**Secoiridoids**
Demethyl elenolic acid	-5.8	-6.3	**Secoiridoids**
3,4-DHPEA-EDA (Oleuropein-aglycone di-aldehyde)	-6.9	-6.2	**Secoiridoids**
Hydroxytyrosol acetate	-6.5	-6.2	**Secoiridoids**
Decarboxymethyl ligstroside aglycone	-6.7	-6.0	**Secoiridoids**
Oleocanthal (Dialdehydic form of decarboxymethyl Ligstroside aglycon)	-6.7	-6.0	**Secoiridoids**
Tyrosol acetate	-6.9	-5.9	**Secoiridoids**
Elenolic acid	-6.0	-5.9	**Secoiridoids**
p-HPEA-EDA	-6.8	-5.8	**Secoiridoids**
Hemiacetal of dialdehydic ligstroside aglycone decarboxymethyl	-6.7	-5.8	**Secoiridoids**
Elenolic acid methylester	-5.8	-5.4	**Secoiridoids**
Elenolic acid dialdehyde	-5.4	-5.4	**Secoiridoids**
DEDA (Decarboxymethyl elenolic acid dialdehyde)	-5.4	-5.1	**Secoiridoids**
Acetal of DEDA (Decarboxymethyl elenolic acid dialdehyde)	-5.2	-5.1	**Secoiridoids**
Dialdehydic elenolic acid decarboxymethyl	-5.2	-4.9	**Secoiridoids**
Dialdehydic elenolic ester decarboxymethyl	-5.3	-4.8	**Secoiridoids**
(E)-β-farnesene	-6.4	-6.5	**Sesquiterpenes**
α-Selinene	-6.3	-6.5	**Sesquiterpenes**
β-Curcumene	-6.7	-6.4	**Sesquiterpenes**
β- Sesquiphellandrene	-6.3	-6.4	**Sesquiterpenes**
Drima-7,9(11)-diene	-6.2	-6.3	**Sesquiterpenes**
(E)2,(Z)4,(E)6-Allofarnesene	-5.9	-6.3	**Sesquiterpenes**
α-copaene	-5.4	-6.3	**Sesquiterpenes**
β-cubebene	-6.6	-6.2	**Sesquiterpenes**
α-Zingiberene	-6.5	-6.2	**Sesquiterpenes**
(Z)-β-farnesene	-6.3	-6.1	**Sesquiterpenes**

(Z)2,(E)4,(E)6-Allofarnesene	-6.1	-6.1	**Sesquiterpenes**
Calarene	-6.1	-6.1	**Sesquiterpenes**
(E)-caryophyllene	-6.0	-6.1	**Sesquiterpenes**
δ-cadinene	-6.0	-6.1	**Sesquiterpenes**
α-trans-bergamotene	-5.9	-6.1	**Sesquiterpenes**
Eremophyllene	-6.1	-6.0	**Sesquiterpenes**
γ-curcumene	-6.5	-5.8	**Sesquiterpenes**
Cyclosativene	-6.1	-5.8	**Sesquiterpenes**
β-elemene	-5.9	-5.8	**Sesquiterpenes**
β-acoradiene	-5.7	-5.7	**Sesquiterpenes**
Longicyclene	-5.6	-5.7	**Sesquiterpenes**
Alloaromadendrene	-6.0	-5.6	**Sesquiterpenes**
γ-Muurolene	-4.7	-5.5	**Sesquiterpenes**
3,4-Dihydroxyphenylglycol	-6.1	-5.6	**Simple Phenols**
3,4,5-Trimethoxybenzoic acid	-5.2	-5.5	**Simple Phenols**
3,4-Dimethoxybenzoic acid	-5.9	-5.4	**Simple Phenols**
Homovanillyl alcohol	-5.8	-5.3	**Simple Phenols**
Hydroxytyrosol	-5.8	-5.3	**Simple Phenols**
2,6-Dimethoxybenzoic acid	-5.7	-5.2	**Simple Phenols**
Syringaldehyde	-5.8	-5.0	**Simple Phenols**
4-Ethylguaiacol	-5.6	-5.0	**Simple Phenols**
4-Vinylguaiacol	-5.6	-5.0	**Simple Phenols**
Tyrosol	-5.6	-5.0	**Simple Phenols**
4-Methylcatechol	-5.5	-4.9	**Simple Phenols**
Catechol	-5.3	-4.9	**Simple Phenols**
4-Ethylphenol	-5.6	-4.8	**Simple Phenols**
4-Vinylphenol	-5.6	-4.8	**Simple Phenols**
4-Hydroxybenzaldehyde	-5.5	-4.6	**Simple Phenols**
o-cresol	-5.3	-4.6	**Simple Phenols**
m-cresol	-5.2	-4.6	**Simple Phenols**
p-cresol	-5.1	-4.4	**Simple Phenols**
Phenol	-4.7	-4.4	**Simple Phenols**
Galactinol	-6.7	-7.4	**Sugar Alcohols**
myo-inositol	-5.7	-5.7	**Sugar Alcohols**
D-Mannitol	-5.7	-5.6	**Sugar Alcohols**
D-(+)-chiro-inositol	-5.6	-5.6	**Sugar Alcohols**
L-Fucose	-5.8	-5.4	**Sugar Alcohols**
D-Fucose	-5.5	-5.2	**Sugar Alcohols**
Adonitol	-5.5	-4.8	**Sugar Alcohols**
Xylitol	-5.5	-4.7	**Sugar Alcohols**
L-(-)-arabitol	-5.7	-4.6	**Sugar Alcohols**
D-(+)-galacturonic acid	-6.4	-5.9	**Sugar Carboxylic Acids**

D-glucuronic acid	-6.2	-5.8	**Sugar Carboxylic Acids**
2-Ethyl-5-hexylthiophene	-5.4	-5.1	**Sulfur Compounds**
2,5-Diethylthiophene	-4.6	-4.2	**Sulfur Compounds**
3-Isopropenylthiophene	-4.7	-4.1	**Sulfur Compounds**
4-Methoxy-2-methyl-2-butanethiol	-3.9	-3.5	**Sulfur Compounds**
3-Methyl-2-butenethiol	-3.6	-3.5	**Sulfur Compounds**
Squalene	-5.6	-6.1	**Terpenic Hydrocarbons**
Eremophilone	-6.1	-5.6	**Terpenic Hydrocarbons**
6,10-Dimethyl-l-undecane	-5.0	-4.7	**Terpenic Hydrocarbons**
α-tocopherol	-7.3	-6.6	**Tocopherols**
δ-tocopherol	-6.9	-6.5	**Tocopherols**
β-tocopherol	-6.9	-6.0	**Tocopherols**
γ-tocopherol	-6.2	-5.8	**Tocopherols**
Maltotriose	-7.0	-8.0	**Triasaccharides**
D-(+)-raffinose	-7.2	-7.4	**Triasaccharides**
L-rhamnose	-5.5	-5.3	**Triasaccharides**
Tirucallol	-8.7	-9.3	**Triterpene Alcohols**
α-amyrin	-8.5	-9.2	**Triterpene Alcohols**
β-amyrin	-8.3	-9.2	**Triterpene Alcohols**
28-hydroxytaraxerol	-8.8	-8.9	**Triterpene Alcohols**
Taraxerol	-8.6	-8.9	**Triterpene Alcohols**
Ψ-taraxasterol	-9.0	-8.8	**Triterpene Alcohols**
Agrostophyllinol	-8.8	-8.7	**Triterpene Alcohols**
Lupeol	-8.8	-8.7	**Triterpene Alcohols**
Butyrospermol	-8.2	-8.7	**Triterpene Alcohols**
δ-amyrin	-8.7	-8.6	**Triterpene Alcohols**
Linalool	-8.2	-8.6	**Triterpene Alcohols**
3-epi-lupeol	-8.7	-8.5	**Triterpene Alcohols**
4α,14α-Dimethylstigmasta-8,24(24)-dien-3β-ol	-8.7	-8.5	**Triterpene Alcohols**

7, 24-tirucalladienol	-8.2	-8.5	**Triterpene Alcohols**
β-amyrone	-8.2	-8.4	**Triterpene Alcohols**
Taraxasterol	-8.1	-8.4	**Triterpene Alcohols**
28-nor-β-amyrin	-8.6	-8.3	**Triterpene Alcohols**
24-methylene-24-dihydroparkeol	-8.3	-8.2	**Triterpene Alcohols**
28-nor-α-amyrin	-8.3	-8.2	**Triterpene Alcohols**
Bacchar-12,21-dien-3β-ol	-7.7	-8.2	**Triterpene Alcohols**
4,4-dimethyl-5α-stigmasta-7,24Z(241)-dien-3β-ol	-8.1	-8.1	**Triterpene Alcohols**
Cycloartenol	-8.1	-8.1	**Triterpene Alcohols**
24-methylene-24-dihydrolanosterol	-8.5	-8.0	**Triterpene Alcohols**
Parkeol	-8.1	-8.0	**Triterpene Alcohols**
Cyclobranol	-8.0	-8.0	**Triterpene Alcohols**
24-methylene-24-dihydroparkenol	-8.7	-7.9	**Triterpene Alcohols**
4,4-dimethyl-5α-stigmast-7-en-3β-ol	-7.9	-7.9	**Triterpene Alcohols**
24-methylene-cycloartenol	-8.2	-7.8	**Triterpene Alcohols**
(24Z)-24-ethylidene-dihydrolanosterol	-8.8	-7.7	**Triterpene Alcohols**
Methyl 2α,3β-diacetoxyolean-12-en-28-oate	-8.4	-7.7	**Triterpene Alcohols**
Germanicol	-7.8	-7.7	**Triterpene Alcohols**
Methyl 3β-acetoxyolean-12-en-28-oate	-8.0	-7.6	**Triterpene Alcohols**
Cyclosadol	-7.8	-7.6	**Triterpene Alcohols**
3-epi-betulin	-8.0	-7.5	**Triterpene Alcohols**
Dammaradienol	-7.8	-7.4	**Triterpene Alcohols**
Lupenone	-6.2	-5.4	**Triterpene Alcohols**
Maslinic acid	-8.7	-9.4	**Triterpenic Acids**
Oleanolic acid	-8.7	-9.1	**Triterpenic Acids**

Urs-2β,3β-dihydroxy-12-en-28-oic acid	-9.1	-9.0	**Triterpenic Acids**
Corosolic acid	-8.9	-8.9	**Triterpenic Acids**
Oleanolic acid demethyl	-8.5	-8.8	**Triterpenic Acids**
Ursolic acid	-8.8	-8.7	**Triterpenic Acids**
Pomolic acid	-8.5	-8.6	**Triterpenic Acids**
3-epi-betulinic acid	-8.9	-8.1	**Triterpenic Acids**
Betulinic acid	-8.9	-8.1	**Triterpenic Acids**
Erythrodiol	-8.4	-8.4	**Triterpenic Dialcohols**
Uvaol	-8.3	-8.3	**Triterpenic Dialcohols**
Geranylgeranyl C20:0	-5.5	-5.6	**Wax Esters**
Geranylgeranyl oleate C18:0	-5.2	-5.6	**Wax Esters**
Geranylgeranyl oleate C18:1	-6.3	-5.5	**Wax Esters**
Geranylgeranyl C24:0	-5.7	-5.5	**Wax Esters**
Geranylgeranyl C20:1	-5.5	-5.5	**Wax Esters**
Phytyl oleate C18:0	-5.8	-5.4	**Wax Esters**
Wax ester 40:0 (20:0-20:0)	-5.3	-5.4	**Wax Esters**
Geranylgeranyl C22:0	-5.5	-5.3	**Wax Esters**
Wax ester 44:1 (20:1-24:0)	-5.4	-5.3	**Wax Esters**
Wax ester 40:1 (18:1-22:0)	-5.1	-5.3	**Wax Esters**
Wax ester 44:0 (22:0-22:0)	-4.5	-5.3	**Wax Esters**
Wax ester 44:1 (16:0-28:0)	-5.0	-5.2	**Wax Esters**
Wax ester 42:0 (24:0-18:0)	-5.3	-5.1	**Wax Esters**
Phytyl oleate C18:1	-5.0	-5.1	**Wax Esters**
Wax ester 40:0 (16:0-24:0)	-4.6	-5.1	**Wax Esters**
Wax ester 40:1 (16:1-24:0)	-5.4	-5.0	**Wax Esters**
Wax ester 46:0 (22:0-24:0)	-5.1	-5.0	**Wax Esters**
Phytyl C22:0	-4.8	-5.0	**Wax Esters**
Wax ester 38:0 (14:0-24:0)	-5.5	-4.9	**Wax Esters**
Wax ester 38:0 (12:0-26:0)	-5.3	-4.9	**Wax Esters**
Phytyl C20:1	-5.0	-4.9	**Wax Esters**
Phytyl C20:0	-4.9	-4.9	**Wax Esters**
Wax ester 42:0 (20:0-22:0)	-4.8	-4.9	**Wax Esters**
Wax ester 38:0 (20:0-18:0)	-4.5	-4.9	**Wax Esters**
Wax ester 42:0 (14:0-28:0)	-4.5	-4.9	**Wax Esters**
Wax ester 38:0 (16:0-22:0)	-5.2	-4.8	**Wax Esters**
Wax ester 42:1 (18:1-24:0)	-5.2	-4.8	**Wax Esters**
Phytyl C24:0	-5.0	-4.8	**Wax Esters**
Wax ester 44:0 (18:0-26:0)	-5.0	-4.8	**Wax Esters**
Ethyl oleate	-5.6	-4.7	**Wax Esters**
Methyl stearate	-5.6	-4.7	**Wax Esters**

Wax ester 42:1 (16:1-26:0)	-5.4	-4.7	**Wax Esters**
Methyl oleate	-5.3	-4.7	**Wax Esters**
Wax ester 42:0 (18:0-24:0)	-5.1	-4.7	**Wax Esters**
Wax ester 46:0 (24:0-22:0)	-5.1	-4.7	**Wax Esters**
Wax ester 46:1 (18:1-28:0)	-5.1	-4.7	**Wax Esters**
Wax ester 42:0 (16:0-26:0)	-5.0	-4.7	**Wax Esters**
Wax ester 46:0 (16:0-30:0)	-4.9	-4.7	**Wax Esters**
Wax ester 40:0 (18:0-22:0)	-4.6	-4.7	**Wax Esters**
Methyl palmitate	-5.2	-4.6	**Wax Esters**
Wax ester 40:0 (14:0-26:0)	-5.1	-4.6	**Wax Esters**
Wax ester 38:0 (18:0-20:0)	-5.1	-4.5	**Wax Esters**
Wax ester 46:0 (14:0-32:0)	-4.4	-4.5	**Wax Esters**
Wax ester 46:0 (20:0-26:0)	-5.2	-4.4	**Wax Esters**
Wax ester 44:1 (18:1-26:0)	-5.1	-4.4	**Wax Esters**
Wax ester 46:0 (18:0-28:0)	-4.5	-4.4	**Wax Esters**
Wax ester 44:0 (16:0-28:0)	-5.0	-4.2	**Wax Esters**
Luteoxanthin	33.7	-9.6	**Xanthophylls**
Neoxanthin	9.1	-9.0	**Xanthophylls**
Mutatoxanthin	24.9	-9.0	**Xanthophylls**
β-cryptoxanthin	-6.6	-8.7	**Xanthophylls**
Violaxanthin	28.7	-8.5	**Xanthophylls**
Antheraxanthin	-6.9	-8.2	**Xanthophylls**

Table S5. Predicted binding affinities (kcal/mol) of the compounds screened against the G-loop region of the GIRK4^{WT} and GIRK4^{G151E} channels.

	**GIRK4^{WT}**	**GIRK4^{G151E}**	
**Compound**	**Binding Affinity (kcal/mol)**	**Binding Affinity (kcal/mol)**	**Classification**
24-Ethyl-E-23-dehydrolophenol	-7.7	-10.2	**4-Methylsterols**
24-Ethylidenelophenol	-6.4	-10.1	**4-Methylsterols**
24-ethyllophenol	-7.7	-10.0	**4-Methylsterols**
24-methyl-(E)-23-dehydrolophenol	-7.5	-9.9	**4-Methylsterols**
24-methyl-24(25)-dehydrolophenol	-7.5	-9.9	**4-Methylsterols**
Citrostadienol	-7.6	-9.8	**4-Methylsterols**
28-isocitrostadienol	-8.0	-9.5	**4-Methylsterols**
Cycloeucalenol	-8.5	-9.4	**4-Methylsterols**
24-Methylenelophenol	-6.4	-9.3	**4-Methylsterols**
Gramisterol	-6.4	-9.3	**4-Methylsterols**
24-methylene-31-nor-9(11)-lanostenol	-7.7	-9.2	**4-Methylsterols**
24-methyl-31-nor-9(11)-lanostenol	-7.5	-9.2	**4-Methylsterols**
Obtusifoliol	-7.4	-8.8	**4-Methylsterols**
Terpineol	-5.2	-6.7	**Alcohols**
Lavendulol	-4.9	-6.2	**Alcohols**
2-Phenylethanol	-4.8	-5.8	**Alcohols**
6-Methyl-5-hepten-3-ol	-4.9	-5.3	**Alcohols**
Benzyl alcohol	-4.6	-5.2	**Alcohols**
Decanol	-5.3	-5.1	**Alcohols**
Nonanol	-5.2	-4.9	**Alcohols**
3-Octenol	-4.9	-4.9	**Alcohols**
2-Octanol	-4.8	-4.9	**Alcohols**
Octanol	-4.8	-4.8	**Alcohols**
2-Heptanol	-4.6	-4.5	**Alcohols**
Heptan-2-ol	-4.6	-4.5	**Alcohols**
Heptanol	-4.4	-4.5	**Alcohols**
4-Hexenol	-4.4	-4.4	**Alcohols**
trans-2-Hexenol	-4.4	-4.3	**Alcohols**
trans-3-Hexenol	-4.4	-4.3	**Alcohols**
cis-2-Hexenol	-4.3	-4.3	**Alcohols**
cis-3-Hexenol	-4.3	-4.3	**Alcohols**
Hexanol	-4.1	-4.2	**Alcohols**
3-Penten-2-ol	-4.0	-4.0	**Alcohols**
2-Penten-1-ol	-4.2	-3.9	**Alcohols**

1-Penten-3-ol	-4.1	-3.9	**Alcohols**
3-Pentanol	-4.0	-3.9	**Alcohols**
2-Methyl-3-butenol	-3.8	-3.9	**Alcohols**
Pentanol	-3.9	-3.8	**Alcohols**
2-Methyl-1-butanol	-3.8	-3.8	**Alcohols**
3-Methyl-1-butanol	-3.8	-3.8	**Alcohols**
Butan-1-ol	-3.6	-3.6	**Alcohols**
Butan-2-ol	-3.6	-3.5	**Alcohols**
1-Propanol	-3.2	-3.2	**Alcohols**
Ethanol	-2.6	-2.6	**Alcohols**
Methanol	-2.1	-2.0	**Alcohols**
trans,trans-2,4-Decadienal	-4.8	-5.7	**Aldehydes**
cis-2-Nonenal	-5.9	-5.4	**Aldehydes**
2,4-Heptadienal	-5.7	-5.4	**Aldehydes**
trans-4,5-Epoxy-trans-2-decenal	-5.3	-5.4	**Aldehydes**
2,4 Hexadienal	-5.9	-5.3	**Aldehydes**
trans-2-Decenal	-5.6	-5.3	**Aldehydes**
Nonanal	-5.5	-5.3	**Aldehydes**
cis-2-Hexenal	-4.7	-5.3	**Aldehydes**
2,4-Decadienal	-5.6	-5.2	**Aldehydes**
Vanillin	-5.2	-5.2	**Aldehydes**
Decanal	-5.3	-5.1	**Aldehydes**
trans-2-Nonenal	-5.3	-5.1	**Aldehydes**
Phenylacetaldehyde	-5.2	-5.0	**Aldehydes**
2-Methylbutanal	-4.7	-5.0	**Aldehydes**
Propanal	-4.9	-4.8	**Aldehydes**
trans-2-Octenal	-5.2	-4.7	**Aldehydes**
cis-2-Pentenal	-4.5	-4.6	**Aldehydes**
trans-2-Heptenal	-4.7	-4.5	**Aldehydes**
Octanal	-4.5	-4.3	**Aldehydes**
2-Methyl-2-butenal	-4.4	-4.3	**Aldehydes**
Hexanal	-4.4	-4.3	**Aldehydes**
Acetaldehyde	-4.2	-4.3	**Aldehydes**
cis-3-Hexenal	-4.3	-4.2	**Aldehydes**
trans-2-Hexenal	-4.4	-4.1	**Aldehydes**
Pentanal	-4.2	-4.1	**Aldehydes**
3-Hexanal	-3.9	-4.1	**Aldehydes**
Heptanal	-3.9	-4.0	**Aldehydes**
trans-2-Pentenal	-4.0	-3.9	**Aldehydes**
3-Methylbutanal	-3.9	-3.8	**Aldehydes**
trans,cis-2,4-Decadienal	-3.8	-3.8	**Aldehydes**
Benzaldehyde	-3.7	-3.8	**Aldehydes**
trans,trans-2,4-Nonadienal	-3.6	-3.7	**Aldehydes**
cis-2-Heptenal	-2.5	-2.4	**Aldehydes**

Dotriacontane	-6.6	-6.9	**Alkanes**
Nonacosane	-6.8	-6.7	**Alkanes**
Tetratriacontane	-6.7	-6.7	**Alkanes**
Tricosane	-6.7	-6.7	**Alkanes**
Tritriacontane	-6.9	-6.6	**Alkanes**
Triacontane	-6.6	-6.6	**Alkanes**
Tetracosane	-6.3	-6.6	**Alkanes**
Hentriacontane	-6.7	-6.5	**Alkanes**
Octacosane	-6.7	-6.5	**Alkanes**
Pentacosane	-6.5	-6.5	**Alkanes**
Hexacosane	-6.4	-6.5	**Alkanes**
Heptacosane	-6.8	-6.4	**Alkanes**
Icosane	-6.8	-6.4	**Alkanes**
Docosane	-6.5	-6.4	**Alkanes**
Heneicosane	-6.5	-6.4	**Alkanes**
Octadecane	-6.5	-6.3	**Alkanes**
Nonadecane	-6.7	-6.2	**Alkanes**
Heptadecane	-6.5	-6.1	**Alkanes**
Hexadecane	-6.1	-6.0	**Alkanes**
Pentadecane	-6.1	-5.8	**Alkanes**
Tetradecane	-6.0	-5.6	**Alkanes**
Styrene	-5.1	-5.6	**Alkanes**
Dodecane	-5.9	-5.5	**Alkanes**
Tridecane	-5.8	-5.4	**Alkanes**
Methyl benzene	-4.2	-5.2	**Alkanes**
Nonane	-5.1	-5.0	**Alkanes**
Octane	-4.8	-4.6	**Alkanes**
Heptane	-4.4	-4.2	**Alkanes**
2-Methylpentane	-4.3	-4.0	**Alkanes**
3-Methylpentane	-4.1	-4.0	**Alkanes**
Hexane	-4.2	-3.9	**Alkanes**
Hexene	-4.2	-3.8	**Alkanes**
2-Methylbutane	-3.8	-3.7	**Alkanes**
9-Hexacosene	-6.8	-6.8	**Alkenes**
8-Heptadecene	-6.7	-6.6	**Alkenes**
9-Tetracosene	-7.0	-6.5	**Alkenes**
9-Pentacosene	-6.9	-6.5	**Alkenes**
9-Tricosene	-6.8	-6.5	**Alkenes**
9-Heptacosene	-6.5	-6.5	**Alkenes**
9-Docosene	-6.6	-6.4	**Alkenes**
6,10-dimethyl-1-undecene	-5.8	-5.9	**Alkenes**
Phenylalanine	-5.5	-6.3	**Amino Acids**
Tyrosine	-5.7	-6.0	**Amino Acids**
Arginine	-5.3	-5.4	**Amino Acids**

Isoleucine	-4.2	-4.7	**Amino Acids**
Glutamine	-4.8	-4.6	**Amino Acids**
Leucine	-4.3	-4.6	**Amino Acids**
Aspargine	-4.5	-4.5	**Amino Acids**
Valine	-4.0	-4.5	**Amino Acids**
Glutamic acid	-4.6	-4.4	**Amino Acids**
Aspartic acid	-4.2	-4.3	**Amino Acids**
Serine	-3.7	-4.3	**Amino Acids**
Alanine	-3.4	-4.0	**Amino Acids**
Piperaquine	-7.4	-10.2	**Antibiotics**
Biltricide	-8.0	-9.5	**Antibiotics**
Lumefantrine	-7.8	-9.5	**Antibiotics**
Pyronaridine	-8.7	-9.4	**Antibiotics**
Ceftriaxone	-7.7	-9.2	**Antibiotics**
Fusidic acid	-4.5	-9.2	**Antibiotics**
Atovaquone	-9.8	-9.0	**Antibiotics**
Moxifloxacin	-7.9	-8.8	**Antibiotics**
Cefditoren Pivoxil	-7.3	-8.8	**Antibiotics**
Cefamandole	-8.2	-8.7	**Antibiotics**
Mefloquine	-8.6	-8.6	**Antibiotics**
Tafenoquine	-8.7	-8.5	**Antibiotics**
Amodiaquine	-7.7	-8.3	**Antibiotics**
Amoxicillin	-7.4	-8.3	**Antibiotics**
Terbinafine	-7.4	-8.2	**Antibiotics**
Artemisinin	-8.4	-8.1	**Antibiotics**
Gemifloxacin	-8.0	-8.1	**Antibiotics**
Levofloxacin	-7.8	-8.1	**Antibiotics**
Quinine	-7.7	-8.1	**Antibiotics**
Ofloxacin	-8.3	-8.0	**Antibiotics**
Ampicillin	-7.7	-8.0	**Antibiotics**
Quinacrine	-7.6	-8.0	**Antibiotics**
Benzylpenicillin	-7.5	-8.0	**Antibiotics**
Doxycycline	-7.2	-8.0	**Antibiotics**
Cefuroxime	-7.9	-7.9	**Antibiotics**
Linezolid	-7.4	-7.9	**Antibiotics**
Cefaclor	-7.9	-7.8	**Antibiotics**
Cefotaxime	-7.8	-7.8	**Antibiotics**
Niclosamide	-7.7	-7.8	**Antibiotics**
Nitazoxanide	-7.3	-7.8	**Antibiotics**
Gatifloxacin	-8.0	-7.7	**Antibiotics**
Ciprofloxacin	-7.8	-7.7	**Antibiotics**
Norfloxacin	-7.4	-7.5	**Antibiotics**
Sulfamethoxazole	-7.2	-7.5	**Antibiotics**
Tizoxanide	-6.8	-7.5	**Antibiotics**

Pyrimethamine	-6.7	-7.5	**Antibiotics**
Hydroxychloroquine	-6.6	-7.4	**Antibiotics**
Chloroquine	-6.3	-7.2	**Antibiotics**
Oxolinic acid	-6.8	-7.0	**Antibiotics**
Trimethoprim	-6.7	-7.0	**Antibiotics**
Chloramphenicol	-6.4	-7.0	**Antibiotics**
Proguanil	-6.1	-6.9	**Antibiotics**
Levamisole	-5.9	-6.6	**Antibiotics**
Clioquinol	-5.8	-6.1	**Antibiotics**
Metronidazole	-4.7	-5.0	**Antibiotics**
Pyrazinamide	-4.4	-4.7	**Antibiotics**
Polymyxin B1	11.7	8.3	**Antibiotics**
Octanoic acid	-5.2	-4.9	**Carboxylic Acids**
Hexanoic acid	-4.6	-4.8	**Carboxylic Acids**
Heptanoic acid	-4.8	-4.6	**Carboxylic Acids**
Pentanoic acid	-4.3	-4.4	**Carboxylic Acids**
3-Methyl-butanoic acid	-4.1	-4.1	**Carboxylic Acids**
3-Methylbutyric acid	-4.1	-4.1	**Carboxylic Acids**
Butanoic acid	-4.0	-3.9	**Carboxylic Acids**
Isobutyric acid	-3.9	-3.9	**Carboxylic Acids**
Propanoic acid	-3.5	-3.7	**Carboxylic Acids**
Acetic acid	-3.1	-3.2	**Carboxylic Acids**
Lutein	-5.8	2.9	**Carotenoids**
β-Carotene	-5.8	10.5	**Carotenoids**
Pheophorbide a	-3.1	-6.5	**Chlorophylls**
Pyropheophytin α	-5.4	-6.0	**Chlorophylls**
Pheophorbide b	-2.9	-5.8	**Chlorophylls**
Chlorophyll b	-1.9	-4.9	**Chlorophylls**
Chlorophyllide a	-2.6	-4.6	**Chlorophylls**
Chlorophyll a	-3.3	-4.4	**Chlorophylls**
Chlorophyllide b	-2.6	-3.1	**Chlorophylls**
Pheophytin b	-1.4	-2.3	**Chlorophylls**
Pheophytin α	0.4	-1.8	**Chlorophylls**
Stigmasterol	-8.7	-10.0	**Common Sterols**
Brassicasterol	-9.0	-9.8	**Common Sterols**
Stigmastanol	-7.7	-9.7	**Common Sterols**

Δ-7-Stigmastenol	-7.5	-9.7	**Common Sterols**
β-sitosterol	-8.1	-9.6	**Common Sterols**
Δ7,24-Ergostadienol	-8.0	-9.6	**Common Sterols**
Δ7,22-Ergostadienol	-8.7	-9.4	**Common Sterols**
Δ-5,24-Stigmastadienol	-8.6	-9.4	**Common Sterols**
Δ-5,23-Stigmastadienol	-8.3	-9.4	**Common Sterols**
Cholesterol	-8.5	-9.3	**Common Sterols**
Ergosterol	-8.3	-9.3	**Common Sterols**
Δ-5-Avenasterol	-8.3	-9.3	**Common Sterols**
24-Methylene-cholesterol	-8.2	-9.3	**Common Sterols**
22,23-Dihydrobrassicasterol	-8.0	-9.3	**Common Sterols**
Clerosterol	-8.1	-9.2	**Common Sterols**
Δ-7-Avenasterol	-7.8	-9.2	**Common Sterols**
Campestanol	-7.7	-9.2	**Common Sterols**
Campesterol	-7.5	-8.7	**Common Sterols**
Esculin	-7.5	-8.5	**Coumarins**
Scopolin	-7.5	-8.3	**Coumarins**
Scopoletin	-6.0	-6.4	**Coumarins**
Esculetin	-5.9	-6.2	**Coumarins**
1,2-diacylglycerol	-4.2	-4.5	**Diacylglycerols**
1,3-diacylglycerol	-4.3	-4.4	**Diacylglycerols**
2,3-diacylglycerol	-4.3	-4.3	**Diacylglycerols**
D-(+)-lactose	-6.1	-6.4	**Diasaccharides**
D-(+)-sucrose	-6.1	-6.1	**Diasaccharides**
Geranylgeraniol	-7.1	-7.7	**Diterpene Alcohols**
Phytol	-6.4	-7.0	**Diterpene Alcohols**
cis-3-Hexenyl acetate	-6.2	-7.6	**Esters**
2-Ethylphenyl acetate	-5.3	-6.4	**Esters**
Phenethyl acetate	-5.4	-6.3	**Esters**
Ethyl cinnamate	-5.9	-6.2	**Esters**
Benzyl acetate	-5.4	-6.0	**Esters**
Ethyl cyclohexylcarboxylate	-5.1	-5.7	**Esters**
Ethyl octanoate	-5.4	-5.2	**Esters**
Octyl acetate	-5.4	-5.2	**Esters**
3-Hexenyl acetate	-5.3	-5.1	**Esters**
Methyl octanoate	-5.3	-5.0	**Esters**
2-Hexenyl acetate	-5.1	-5.0	**Esters**
2-Methylpropyl butanoate	-4.7	-5.0	**Esters**
Hexyl acetate	-4.9	-4.8	**Esters**
Methyl heptanoate	-4.9	-4.8	**Esters**
Ethyl hexanoate	-4.8	-4.7	**Esters**

Isopentyl acetate	-4.6	-4.7	**Esters**
2-Methylbutyl acetate	-4.3	-4.6	**Esters**
Ethyl 3-methylbutanoate	-4.3	-4.6	**Esters**
Methyl hexanoate	-4.6	-4.5	**Esters**
Propyl butanoate	-4.5	-4.5	**Esters**
Propyl 2-methylpropanoate	-4.4	-4.5	**Esters**
Ethyl 2-methylbutanoate	-4.2	-4.5	**Esters**
Methyl 3-methylbutanoate	-4.0	-4.3	**Esters**
Methyl pentanoate	-4.2	-4.2	**Esters**
Ethyl isobutyrate	-4.1	-4.2	**Esters**
Propyl propanoate	-4.2	-4.1	**Esters**
Ethyl butanoate	-4.1	-4.1	**Esters**
Ethyl-2-methypropanoate	-4.1	-4.1	**Esters**
Methyl 2-methylbutanoate	-3.9	-4.1	**Esters**
Butyl acetate	-4.4	-4.0	**Esters**
Ethyl propanoate	-3.9	-4.0	**Esters**
Methyl butanoate	-3.8	-3.9	**Esters**
Ethyl acetate	-3.7	-3.7	**Esters**
Methyl acetate	-3.2	-3.4	**Esters**
1,8-Cineole	-5.3	-6.2	**Ethers**
Diethyl ether	-3.1	-3.2	**Ethers**
Ethyl linoleate	-6.3	-7.0	**Ethyl Esters**
Ethyl oleate	-7.1	-6.7	**Ethyl Esters**
Ethyl stearate	-6.4	-6.5	**Ethyl Esters**
Ethyl palmitate	-6.8	-6.3	**Ethyl Esters**
Hexacosanol	-6.7	-6.7	**Fatty Alcohols**
Pentacosanol	-6.5	-6.7	**Fatty Alcohols**
Heptacosanol	-6.4	-6.7	**Fatty Alcohols**
Docosanol	-6.6	-6.6	**Fatty Alcohols**
Octacosanol	-6.5	-6.5	**Fatty Alcohols**
Tetracosanol	-6.1	-6.5	**Fatty Alcohols**
Eicosanol	-6.6	-6.3	**Fatty Alcohols**
Tricosanol	-6.6	-6.2	**Fatty Alcohols**
Silibinin	-8.9	-10.6	**Flavonoids**
Apigenin-7-O-rutinoside	-8.3	-10.5	**Flavonoids**
Luteolin-7-O-rutinoside	-6.6	-10.4	**Flavonoids**
Flavoxate	-8.8	-10.2	**Flavonoids**
Diosmin	-7.9	-10.1	**Flavonoids**
Hesperidin	-7.4	-10.1	**Flavonoids**
alpha-Naphthoflavone	-9.1	-10.0	**Flavonoids**
Luteolin-4’-O-glucoside	-9.4	-9.9	**Flavonoids**
Sofalcone	-8.8	-9.9	**Flavonoids**
Icariin	-6.0	-9.8	**Flavonoids**
Icaritin	-8.6	-9.7	**Flavonoids**

beta-Naphthoflavone	-8.5	-9.7	**Flavonoids**
Hidrosmin	-6.6	-9.7	**Flavonoids**
Quercetin 3-O-rutinoside	-8.4	-9.6	**Flavonoids**
Rutin	-7.3	-9.6	**Flavonoids**
Luteolin-3′,7-O-diglucoside	-7.2	-9.6	**Flavonoids**
Luteolin-6-C-glucoside	-7.8	-9.5	**Flavonoids**
Luteolin-4'-O-rutinoside	-8.0	-9.4	**Flavonoids**
Lucidumoside C	-7.8	-9.4	**Flavonoids**
Monoxerutin	-6.8	-9.4	**Flavonoids**
Daidzin	-8.3	-9.3	**Flavonoids**
Scolymoside	-7.7	-9.3	**Flavonoids**
Elafibranor	-7.5	-9.3	**Flavonoids**
Chrysoeriol-7-O-glucoside	-9.0	-9.1	**Flavonoids**
Epigallocatechin gallate	-8.8	-9.1	**Flavonoids**
Cyanidin-3-O-glucoside	-7.8	-9.1	**Flavonoids**
Vicenin-2	-6.3	-9.1	**Flavonoids**
Luteolin-7-O-glucoside	-9.2	-9.0	**Flavonoids**
ME-344	-8.9	-9.0	**Flavonoids**
Triphendiol	-8.2	-9.0	**Flavonoids**
Cyanidin-3-O-rutinoside	-8.1	-9.0	**Flavonoids**
Crofelemer	-8.0	-9.0	**Flavonoids**
Apigenin trimethylether	-9.2	-8.9	**Flavonoids**
Acacetin diacetate	-8.4	-8.9	**Flavonoids**
Recoflavone	-8.0	-8.9	**Flavonoids**
Efloxate	-7.9	-8.8	**Flavonoids**
Ipriflavone	-7.7	-8.8	**Flavonoids**
Quercitrin	-6.8	-8.7	**Flavonoids**
Luteolin-8-C-glucoside	-8.9	-8.6	**Flavonoids**
Quercetin-3-O-glucoside	-7.8	-8.6	**Flavonoids**
Flavone	-7.3	-8.6	**Flavonoids**
Troxerutin	-6.7	-8.6	**Flavonoids**
Isoquercetin	-8.3	-8.5	**Flavonoids**
4-methylumbelliferyl beta-D-glucoside	-7.9	-8.5	**Flavonoids**
Baicalein	-7.8	-8.5	**Flavonoids**
Delphinidin-3-O-glucoside	-7.2	-8.5	**Flavonoids**
Acacetin	-7.8	-8.4	**Flavonoids**
Isoflavone	-7.1	-8.4	**Flavonoids**
Luteolin-7,4-O-diglucoside	-5.4	-8.4	**Flavonoids**
4'-Hydroxyflavanone	-7.7	-8.3	**Flavonoids**
Diosmetin	-7.7	-8.3	**Flavonoids**
Idronoxil	-7.7	-8.3	**Flavonoids**
(2S)-7-hydroxyflavanone	-7.4	-8.3	**Flavonoids**

Fisetin	-7.3	-8.3	**Flavonoids**
Quercetin-3'-O-phosphate	-8.6	-8.2	**Flavonoids**
Isoformononetin	-8.0	-8.2	**Flavonoids**
Apigenin-7-O-glucoside	-7.9	-8.2	**Flavonoids**
Chrysin	-7.7	-8.2	**Flavonoids**
Equol	-7.7	-8.2	**Flavonoids**
Hesperetin	-7.6	-8.2	**Flavonoids**
Naringenin	-7.6	-8.2	**Flavonoids**
PD-98059	-7.6	-8.2	**Flavonoids**
Cyanidin (cation)	-7.3	-8.2	**Flavonoids**
Genkwanin	-7.7	-8.1	**Flavonoids**
Hesperitin	-7.7	-8.1	**Flavonoids**
5-deoxyflavanone	-7.6	-8.1	**Flavonoids**
Apigenin	-7.6	-8.1	**Flavonoids**
Chrysoeriol	-7.6	-8.1	**Flavonoids**
Cianidanol	-7.5	-8.1	**Flavonoids**
Hispidulin	-7.4	-8.1	**Flavonoids**
Sakuranetin	-7.7	-8.0	**Flavonoids**
Luteolin	-7.6	-8.0	**Flavonoids**
Eupatilin	-7.4	-8.0	**Flavonoids**
Eriodictyol	-8.1	-7.9	**Flavonoids**
Formononetin	-8.0	-7.9	**Flavonoids**
Methoxyluteolin	-7.6	-7.9	**Flavonoids**
7-(alpha-D-Glucopyranosyloxy)-4-methyl-2H-1-benzopyran-2-one	-7.5	-7.9	**Flavonoids**
Genistein	-7.6	-7.8	**Flavonoids**
Tricetin	-7.5	-7.8	**Flavonoids**
Dihydroxymethoxychalcone	-7.0	-7.8	**Flavonoids**
Biochanin A	-7.8	-7.7	**Flavonoids**
Isorhamnetin	-7.7	-7.7	**Flavonoids**
Kaempherol	-7.6	-7.7	**Flavonoids**
Taxifolin	-7.6	-7.7	**Flavonoids**
Rhamnetin	-7.6	-7.6	**Flavonoids**
Epigallocatechin	-7.4	-7.6	**Flavonoids**
5,7,2'-trihydroxy-6,8-dimethoxyflavone	-7.3	-7.6	**Flavonoids**
Phloretin	-6.7	-7.6	**Flavonoids**
Quercetin	-7.5	-7.5	**Flavonoids**
Delphinidin	-7.4	-7.5	**Flavonoids**
Morin	-7.6	-7.4	**Flavonoids**
Dihydromyricetin	-7.4	-7.4	**Flavonoids**
Epicatechin	-7.3	-7.4	**Flavonoids**

Myricetin	-7.4	-7.3	**Flavonoids**
3,4-methyl-3-pentenyl furan	-5.5	-6.0	**Furans**
3-Methyl-2-pentylfuran	-5.4	-5.8	**Furans**
2-Propylfuran	-4.4	-4.7	**Furans**
3-Propylfuran	-4.4	-4.6	**Furans**
2-Ethylfuran	-4.1	-4.3	**Furans**
Isoverbascoside	-8.6	-10.3	**Glucosides**
Oxidized verbacoside	-8.0	-10.0	**Glucosides**
Actoside	-7.7	-10.0	**Glucosides**
Oxidized isoverbascoside	-8.3	-9.9	**Glucosides**
Hellicoside	-8.3	-9.8	**Glucosides**
Verbascoside	-7.7	-9.8	**Glucosides**
Isoacteoside	-7.9	-9.6	**Glucosides**
Comselogoside	-7.7	-9.4	**Glucosides**
Quercetin-3-rhamnoside	-6.6	-9.4	**Glucosides**
4' -O-β-D-Glucosyl-9-O-(6''-deoxysaccharosyl)olivil	-4.2	-9.4	**Glucosides**
Orbanchoside	-8.4	-9.3	**Glucosides**
Suspensaside	-7.5	-9.3	**Glucosides**
Caffeoyl-6'-secologanoside	-8.1	-9.0	**Glucosides**
Hydroxytyrosol diglucoside	-7.7	-8.9	**Glucosides**
Quercetin-7-O-glucoside	-7.6	-8.7	**Glucosides**
Verucosin	-8.4	-8.4	**Glucosides**
6'-Rhamnopyranosyl oleoside	-7.3	-8.4	**Glucosides**
6'-β-D-Glucopyranosyl oleoside	-7.1	-8.4	**Glucosides**
Wedelosin	-2.7	-8.2	**Glucosides**
6'-O-[(2E)-2,6-Dimethyl-8-hydroxy- 2-octenoyloxy]-secologanoside	-6.8	-8.1	**Glucosides**
Oleoside-11-Methylester	-7.6	-7.6	**Glucosides**
Oleoside	-7.5	-7.6	**Glucosides**
Hydroxytyrosol rhamnoside	-7.0	-7.4	**Glucosides**
Hydroxytyrosol-1′-β-glucoside	-6.9	-7.4	**Glucosides**
Salidroside	-6.7	-7.4	**Glucosides**
Hydroxytyrosol-4-β-glucoside	-6.6	-7.2	**Glucosides**
Secologanoside	-7.3	-7.1	**Glucosides**

Hydroxytyrosol-3-β-glucoside	-6.9	-6.6	**Glucosides**
Phloretic acid	-5.8	-6.3	**Hydroxybenzoic Acids**
2,3-dihydrocaffeic acid	-5.9	-6.2	**Hydroxybenzoic Acids**
Gallic acid	-5.5	-5.6	**Hydroxybenzoic Acids**
4-hydroxybenzoic acid	-4.9	-5.6	**Hydroxybenzoic Acids**
Protocatechuic acid	-5.4	-5.5	**Hydroxybenzoic Acids**
Syringic acid	-5.3	-5.5	**Hydroxybenzoic Acids**
Vanillic acid	-5.2	-5.5	**Hydroxybenzoic Acids**
Quinic acid	-5.1	-5.5	**Hydroxybenzoic Acids**
Shikimic acid	-5.1	-5.5	**Hydroxybenzoic Acids**
4-O-methyl-D-glucuronic acid	-5.3	-5.4	**Hydroxybenzoic Acids**
2,4 dihydroxybenzoic acid	-5.2	-5.4	**Hydroxybenzoic Acids**
Gentisic acid	-5.2	-5.4	**Hydroxybenzoic Acids**
2,6-Dihydroxybenzoic acid	-5.2	-5.2	**Hydroxybenzoic Acids**
β-Hydroxy verbascoside	-8.0	-9.8	**Hydroxycinnamic Acids**
Chlorogenic acid	-7.7	-8.4	**Hydroxycinnamic Acids**
Rosmarinic acid	-7.9	-8.3	**Hydroxycinnamic Acids**
Caffeoylglucose	-7.7	-8.0	**Hydroxycinnamic Acids**
Caftaric acid	-7.1	-7.4	**Hydroxycinnamic Acids**
Ferulic acid	-6.0	-6.3	**Hydroxycinnamic Acids**
Dihydro-p-coumaric acid	-5.8	-6.3	**Hydroxycinnamic Acids**
Cinnamic acid	-5.6	-6.1	**Hydroxycinnamic Acids**
o-Coumaric acid	-5.6	-6.1	**Hydroxycinnamic Acids**
Hydroxycaffeic acid	-6.0	-6.0	**Hydroxycinnamic Acids**

m-Coumaric acid	-5.6	-6.0	**Hydroxycinnamic Acids**
p-Coumaric acid	-5.6	-6.0	**Hydroxycinnamic Acids**
Caffeic acid	-5.9	-5.9	**Hydroxycinnamic Acids**
Sinapic acid	-6.2	-5.8	**Hydroxycinnamic Acids**
1-(3’-Methoxy-4’-hydroxy)- phenyl-6,7-dihydroxyisochroman	-6.8	-7.6	**Hydroxy-Isochromans**
1-Phenyl-6,7-dihydroxyisochroman	-7.1	-7.3	**Hydroxy-Isochromans**
Homovanillic acid	-5.6	-6.0	**Hydroxyphenyl acetic Acids**
4-Hydroxy-3-methoxy-phenylacetic acid	-5.4	-6.0	**Hydroxyphenyl acetic Acids**
Homoveratric acid	-5.5	-5.9	**Hydroxyphenyl acetic Acids**
p-Hydroxyphenylacetic acid	-5.4	-5.9	**Hydroxyphenyl acetic Acids**
3,4-Dihydroxyphenylacetic acid	-5.4	-5.8	**Hydroxyphenyl acetic Acids**
2,5-Dihydroxyphenylacetic acid	-5.1	-5.8	**Hydroxyphenyl acetic Acids**
Loganic acid	-7.3	-7.4	**Iridoids**
Loganin	-7.5	-7.2	**Iridoids**
trans-β-Damascenone	-6.9	-7.4	**Ketones**
Acetophenone	-4.8	-5.6	**Ketones**
6-Methyl-5-hepten-2-one	-5.0	-5.2	**Ketones**
2-Nonanone	-5.2	-5.1	**Ketones**
cis-1,5-Octadien-3-one	-5.2	-5.0	**Ketones**
3-Octanone	-4.9	-5.0	**Ketones**
1-Octen-3-one	-4.9	-4.9	**Ketones**
2-Octanone	-4.9	-4.8	**Ketones**
Octan-2-one	-4.9	-4.8	**Ketones**
Heptan-2-one	-4.6	-4.5	**Ketones**
2-Heptanone	-4.5	-4.5	**Ketones**
4-Methyl-2-pentanone	-4.1	-4.2	**Ketones**
2-Hexanone	-4.3	-4.1	**Ketones**
1-Penten-3-one	-4.0	-4.1	**Ketones**
3-Pentanone	-3.8	-4.1	**Ketones**
3-Methyl-2-butanone	-3.9	-3.8	**Ketones**
2-Butanone	-3.6	-3.7	**Ketones**
Butan-2-one	-3.6	-3.7	**Ketones**

(+)-Fraxiresinol-1-β-D-glucopyranoside	-7.2	-9.3	**Lignans**
(+)-1-Acetoxypinoresinol-4'-β-D-glucopyranoside	-4.1	-9.3	**Lignans**
1-Acetoxypinoresinol	-8.0	-8.6	**Lignans**
Pinoresinol	-7.9	-8.3	**Lignans**
Berchemol	-8.1	-8.2	**Lignans**
(+)-1-Acetoxypinoresinol-4''-O-methyl ether	-7.9	-8.2	**Lignans**
3-Acetyloxy berchemol	-7.2	-8.2	**Lignans**
(-)-Olivil	-7.5	-8.0	**Lignans**
Syringaresinol	-7.5	-8.0	**Lignans**
(+)-1-Acetoxypinoresinol-4'-β-D-glucopyranoside-4''-O-methyl ether	-4.2	-7.8	**Lignans**
(+)-1-Hydroxypinoresinol-4''-O-methyl ether	-4.2	-7.8	**Lignans**
D-(+)-Erythro-1-(4-hydroxy-3-methoxy)- 214 - phenyl-1,2,3-propantriol	-7.4	-7.7	**Lignans**
(+)-1-Hydroxypinoresinol-4'-β-D-glucopyranoside	-5.1	-7.7	**Lignans**
Hydroxypinoresinol	-5.6	-5.9	**Lignans**
Linolenic	-7.1	-7.3	**Long Chain Fatty Acids**
Erucic	-7.1	-7.2	**Long Chain Fatty Acids**
Lignoceric	-6.5	-7.0	**Long Chain Fatty Acids**
Linoelaidic	-7.2	-6.9	**Long Chain Fatty Acids**
Gadoleic	-6.7	-6.9	**Long Chain Fatty Acids**
Linoleic	-7.0	-6.8	**Long Chain Fatty Acids**
Arachidic	-6.4	-6.8	**Long Chain Fatty Acids**
Oleic	-7.0	-6.7	**Long Chain Fatty Acids**
Trans-palmitoleic	-6.9	-6.7	**Long Chain Fatty Acids**
Eicosenoic	-6.8	-6.7	**Long Chain Fatty Acids**
Behenic	-6.0	-6.7	**Long Chain Fatty Acids**
Elaidic	-7.0	-6.6	**Long Chain Fatty Acids**

11-*cis*-vaccenic	-6.9	-6.6	**Long Chain Fatty Acids**
Petroselinic	-6.8	-6.6	**Long Chain Fatty Acids**
Cis-Heptadecenoic	-6.7	-6.4	**Long Chain Fatty Acids**
Palmitic	-6.6	-6.4	**Long Chain Fatty Acids**
Palmitoleic	-6.6	-6.4	**Long Chain Fatty Acids**
Stearic	-6.6	-6.4	**Long Chain Fatty Acids**
Margaric acid	-6.5	-6.2	**Long Chain Fatty Acids**
Myristic	-6.1	-6.0	**Long Chain Fatty Acids**
Clindamycin	-6.3	-7.5	**Macrolides**
Azithromycin	5.7	-7.3	**Macrolides**
Erythromycin C	2.2	-7.1	**Macrolides**
Erythromycin A oxime	3.4	-6.7	**Macrolides**
Pseudo erythromycin A enol ether	-3.7	-5.7	**Macrolides**
Anhydroerythromycin A	-0.5	-5.5	**Macrolides**
Erythromycin	2.6	-5.5	**Macrolides**
N-demethyl roxithromycin	2.6	-5.3	**Macrolides**
Roxithromycin D7	3.1	-5.3	**Macrolides**
Idremcinal	2.5	-5.2	**Macrolides**
Erythromycin B	4.0	-5.2	**Macrolides**
Clarithromycin	1.7	-4.7	**Macrolides**
Decladinose roxithromycin	-1.9	-4.2	**Macrolides**
Telithromycin	3.2	-3.7	**Macrolides**
Josamycin	3.5	-3.7	**Macrolides**
Troleandomycin	3.6	-2.5	**Macrolides**
Flurithromycin	5.3	-2.2	**Macrolides**
Ivermectin	19.6	-1.7	**Macrolides**
Roxithromycin	5.5	-1.2	**Macrolides**
Dirithromycin	4.9	3.2	**Macrolides**
Rapamycin	29.8	33.7	**Macrolides**
Lauric	-5.7	-5.6	**Medium Chain Fatty Acids**
Isoeugenol	-5.7	-6.3	**Methoxyphenols**
2-Methoxy-4-vinylphenol	-5.4	-5.7	**Methoxyphenols**
Homovanillin	-5.3	-5.7	**Methoxyphenols**
Guaiacol	-4.8	-4.9	**Methoxyphenols**
Methyl linoleate	-7.0	-7.0	**Methyl Esters**
Methyl stearate	-6.8	-6.5	**Methyl Esters**

Methyl oleate	-6.6	-6.5	**Methyl Esters**
Methyl heptadecanoate	-6.6	-6.3	**Methyl Esters**
Methyl palmitate	-6.5	-6.3	**Methyl Esters**
1-monoacylglycerol	-5.1	-4.4	**Monocylglycerols**
3-monoacylglycerol	-5.2	-4.3	**Monocylglycerols**
2-monoacylglycerol	-5.0	-4.0	**Monocylglycerols**
Sedoheptulose	-5.7	-5.5	**Monosaccharides**
D-(-)-fructose	-5.1	-5.5	**Monosaccharides**
D-(+)-xylose	-4.8	-5.3	**Monosaccharides**
D-(+)-glucose	-5.2	-5.1	**Monosaccharides**
1,6-anhydro-β-D-glucose	-4.7	-5.1	**Monosaccharides**
D-(+)-mannose	-5.3	-5.0	**Monosaccharides**
D-(-)-arabinose	-4.7	-4.9	**Monosaccharides**
D-(-)-galactose	-5.3	-4.8	**Monosaccharides**
Mannan	-5.9	-6.7	**Oligosaccharides**
α-Cellulose	-6.2	-6.4	**Oligosaccharides**
Galacturonan	-5.2	-5.5	**Oligosaccharides**
Pectin	-5.5	-5.3	**Oligosaccharides**
Citric acid	-4.8	-5.0	**Organic Acids**
Gluconic acid	-4.5	-4.6	**Organic Acids**
Succinic acid	-4.1	-4.6	**Organic Acids**
Malic acid	-4.5	-4.5	**Organic Acids**
Oxalic acid	-3.6	-4.0	**Organic Acids**
Poly-unsaturated di-galactoside glycerol diester	-7.5	-9.1	**Other**
3-[1-(hydroxymethyl)-(E)-1-propenyl] glutaric acid	-6.3	-6.5	**Other**
3-(1-Hydroxymethyl-1-propenyl)pentanedioic acid	-5.2	-5.7	**Other**
3-[1-(formyl)-(E)-1-propenyl] glutaric acid	-5.1	-5.5	**Other**

Halleridone	-5.0	-5.5	**Other**
1,5-anhydroxylitol	-4.2	-4.7	**Other**
Deoxyloganic acid lauryl ester	-7.3	-7.9	**Phenolic Fatty Acid Esters**
1-oleyltyrosol	-7.0	-7.7	**Phenolic Fatty Acid Esters**
Lysophosphatidylethanolamine	-7.1	-7.3	**Phospholipids**
Lysophosphatidic acid	-7.3	-7.2	**Phospholipids**
Phosphatidylinositol	-6.0	-5.8	**Phospholipids**
Phosphatidylcholine	-5.3	-5.0	**Phospholipids**
Phosphatidylglycerol	-5.1	-5.0	**Phospholipids**
Phosphatidic acid	-4.6	-4.5	**Phospholipids**
Phosphatidylethanolamine	-4.9	-4.4	**Phospholipids**
Demethylligstroside	-8.2	-9.6	**Secoiridoids**
Oleuricine A	-7.9	-9.6	**Secoiridoids**
Jaspolyoside	-4.3	-9.6	**Secoiridoids**
Isojaspolyoside A	-3.9	-9.6	**Secoiridoids**
Oleuropein-3'-O-β-D-glucopyranoside	-8.3	-9.5	**Secoiridoids**
Ligstroside-3'-O-β-D-glucopyranoside	-8.2	-9.5	**Secoiridoids**
Demethyloleuropein	-8.4	-9.4	**Secoiridoids**
Ligstroside derivative 3	-7.2	-9.4	**Secoiridoids**
Neo-nüzhenide	-7.2	-9.4	**Secoiridoids**
Oleuropein-3"-Methyl ether	-8.6	-9.3	**Secoiridoids**
Nüzhenide	-7.0	-9.3	**Secoiridoids**
Ligstroside	-7.5	-9.2	**Secoiridoids**
Oleuricine B	-7.2	-9.2	**Secoiridoids**
Jaspolyanoside	-4.5	-9.0	**Secoiridoids**
10-Hydroxyoleuropein	-8.1	-8.9	**Secoiridoids**
Oleuropein diglucoside	-7.0	-8.9	**Secoiridoids**
Ligstroside derivative 4	-3.8	-8.9	**Secoiridoids**
Oleuroside-10-carboxylic acid	-8.6	-8.8	**Secoiridoids**
7"-S-Hydroxyoleuropein	-7.9	-8.8	**Secoiridoids**
Ligstroside derivative 5	-2.6	-8.8	**Secoiridoids**
Oleuroside	-7.6	-8.7	**Secoiridoids**
Oleuropein	-8.3	-8.6	**Secoiridoids**
Dihydro-oleuropein	-7.8	-8.6	**Secoiridoids**
Ligstroside derivative 2	-7.5	-8.6	**Secoiridoids**
Elenolic acid diglucoside	-6.5	-8.5	**Secoiridoids**
Oleuropeindial Lactone (Cannizzaro-like product)	-7.7	-8.3	**Secoiridoids**

Demethyloleuropein aglycone	-7.1	-8.3	**Secoiridoids**
Ligstroside derivative 1	-8.5	-8.2	**Secoiridoids**
Ligstroside aglycone	-7.2	-8.1	**Secoiridoids**
3,4-DHPEA-DETA	-7.2	-8.0	**Secoiridoids**
Oleuropein aglycone (3,4-DHPEA-EA)	-7.7	-7.9	**Secoiridoids**
Ligstroside aglycone methyl acetal	-6.9	-7.9	**Secoiridoids**
7-Deoxyloganic acid	-7.3	-7.8	**Secoiridoids**
Oleuropeindial (enol form)	-7.3	-7.8	**Secoiridoids**
10-Hydroxy oleuropein aglycone decarboxymethyl	-7.7	-7.7	**Secoiridoids**
Nüzhenide oleoside	0.6	-7.7	**Secoiridoids**
10-Hydroxy oleuropein aglycone	-7.8	-7.6	**Secoiridoids**
10-Hydroxy-10-methyl oleuropein aglycone	-7.6	-7.6	**Secoiridoids**
Monoaldehydic form of Ligstroside aglycon	-7.3	-7.6	**Secoiridoids**
Oleoside dimethylester	-7.2	-7.6	**Secoiridoids**
Monoaldehydic form of Oleuropein aglycon	-7.4	-7.5	**Secoiridoids**
Hemiacetal of dialdehydic ligstroside aglycone decarboxymethyl	-6.7	-7.5	**Secoiridoids**
Elenolic acid glucoside	-7.2	-7.4	**Secoiridoids**
p-HPEA-EDA	-6.5	-7.4	**Secoiridoids**
3,4-DHPEA-EDA (Oleuropein-aglycone di-aldehyde)	-6.0	-7.4	**Secoiridoids**
Oleuropein dimer	-2.7	-7.4	**Secoiridoids**
Hydroxytyrosil elenolate	-7.4	-7.3	**Secoiridoids**
demethyloleuropein aglycone (enol form)	-6.7	-7.3	**Secoiridoids**
Decarboxymethyl ligstroside aglycone	-6.4	-7.2	**Secoiridoids**
Nüzhenide 11-Methyl oleoside	-0.2	-7.2	**Secoiridoids**
Oleocanthal (Dialdehydic form of decarboxymethyl Ligstroside aglycon)	-6.7	-7.1	**Secoiridoids**
Secologanin	-6.7	-7.1	**Secoiridoids**
Cornoside	-6.6	-7.0	**Secoiridoids**
Oleacein (Dialdehydic form of decarboxymethyl Oleuropein aglycon)	-6.0	-7.0	**Secoiridoids**

Oleuropeindial (Cannizzaro-like product of oleuropeindial)	-7.5	-6.9	**Secoiridoids**
Secologanic acid	-6.8	-6.9	**Secoiridoids**
Oleuropeindial (keto form)	-7.3	-6.7	**Secoiridoids**
Hemiacetal of dialdehydic oleuropein aglycone decarboxymethyl	-7.0	-6.7	**Secoiridoids**
Demethyloleuropein aglycone dialdehyde	-6.5	-6.7	**Secoiridoids**
Demethyl elenolic acid	-5.8	-6.6	**Secoiridoids**
Secologanol	-6.6	-6.5	**Secoiridoids**
Methyl malate-hydroxytyrosol ester	-6.4	-6.5	**Secoiridoids**
Tyrosol acetate	6.1	-6.5	**Secoiridoids**
Elenolic acid methylester	-5.9	-6.4	**Secoiridoids**
Hydroxytyrosol acetate	-6.1	-6.1	**Secoiridoids**
Elenolic acid	-5.9	-5.9	**Secoiridoids**
Elenolic acid dialdehyde	-5.5	-5.8	**Secoiridoids**
Acetal of DEDA (Decarboxymethyl elenolic acid dialdehyde)	-5.3	-5.7	**Secoiridoids**
Dialdehydic elenolic ester decarboxymethyl	-5.0	-5.6	**Secoiridoids**
DEDA (Decarboxymethyl elenolic acid dialdehyde)	-4.9	-5.4	**Secoiridoids**
Dialdehydic elenolic acid decarboxymethyl	-4.9	-5.4	**Secoiridoids**
(Z)-β-farnesene	-7.3	-9.2	**Sesquiterpenes**
(E)-caryophyllene	-6.7	-9.0	**Sesquiterpenes**
(E)2,(Z)4,(E)6-Allofarnesene	-6.9	-8.7	**Sesquiterpenes**
(E)-β-farnesene	-6.7	-8.7	**Sesquiterpenes**
Calarene	-6.9	-8.6	**Sesquiterpenes**
α-Zingiberene	-7.3	-8.5	**Sesquiterpenes**
β- Sesquiphellandrene	-6.9	-8.5	**Sesquiterpenes**
Drima-7,9(11)-diene	-6.7	-8.5	**Sesquiterpenes**
Alloaromadendrene	-6.6	-8.5	**Sesquiterpenes**
Cyclosativene	-6.9	-8.4	**Sesquiterpenes**
α-Selinene	-6.7	-8.3	**Sesquiterpenes**
α-trans-bergamotene	-6.6	-8.3	**Sesquiterpenes**
β-elemene	-6.9	-8.2	**Sesquiterpenes**
β-Curcumene	-6.8	-8.1	**Sesquiterpenes**
Eremophyllene	-6.8	-8.0	**Sesquiterpenes**
δ-cadinene	-6.7	-8.0	**Sesquiterpenes**
(Z)2,(E)4,(E)6-Allofarnesene	-6.3	-7.7	**Sesquiterpenes**

α-copaene	-6.7	-7.6	**Sesquiterpenes**
γ-curcumene	-6.5	-7.3	**Sesquiterpenes**
β-acoradiene	-6.3	-7.3	**Sesquiterpenes**
β-cubebene	-6.0	-7.3	**Sesquiterpenes**
Longicyclene	-6.6	-7.0	**Sesquiterpenes**
γ-Muurolene	-6.5	-6.7	**Sesquiterpenes**
4-Ethylguaiacol	-5.1	-5.8	**Simple Phenols**
4-Vinylguaiacol	-5.4	-5.7	**Simple Phenols**
3,4,5-Trimethoxybenzoic acid	-5.2	-5.7	**Simple Phenols**
Hydroxytyrosol	-5.4	-5.6	**Simple Phenols**
3,4-Dimethoxybenzoic acid	-5.1	-5.6	**Simple Phenols**
4-Vinylphenol	-5.3	-5.5	**Simple Phenols**
Homovanillyl alcohol	-5.3	-5.5	**Simple Phenols**
4-Ethylphenol	-5.2	-5.5	**Simple Phenols**
2,6-Dimethoxybenzoic acid	-5.0	-5.5	**Simple Phenols**
Tyrosol	-5.0	-5.5	**Simple Phenols**
Syringaldehyde	-5.1	-5.4	**Simple Phenols**
3,4-Dihydroxyphenylglycol	-5.4	-5.3	**Simple Phenols**
4-Methylcatechol	-5.1	-5.3	**Simple Phenols**
m-cresol	-4.8	-5.2	**Simple Phenols**
o-cresol	-4.7	-5.2	**Simple Phenols**
4-Hydroxybenzaldehyde	-5.0	-5.1	**Simple Phenols**
p-cresol	-4.8	-5.1	**Simple Phenols**
Catechol	-4.7	-4.7	**Simple Phenols**
Phenol	-4.4	-4.6	**Simple Phenols**
Galactinol	-6.3	-6.9	**Sugar Alcohols**
D-(+)-chiro-inositol	-5.2	-5.6	**Sugar Alcohols**
myo-inositol	-5.0	-5.4	**Sugar Alcohols**
L-Fucose	-4.7	-5.2	**Sugar Alcohols**
D-Fucose	-5.0	-5.0	**Sugar Alcohols**
Xylitol	-4.9	-4.8	**Sugar Alcohols**
L-(-)-arabitol	-4.2	-4.6	**Sugar Alcohols**
D-Mannitol	-4.5	-4.4	**Sugar Alcohols**
Adonitol	-4.9	-4.2	**Sugar Alcohols**
D-glucuronic acid	-5.1	-5.5	**Sugar Carboxylic Acids**
D-(+)-galacturonic acid	-5.3	-5.1	**Sugar Carboxylic Acids**
2-Ethyl-5-hexylthiophene	-5.8	-6.0	**Sulfur Compounds**

2,5-Diethylthiophene	-4.4	-5.2	**Sulfur Compounds**
3-Isopropenylthiophene	-4.2	-4.9	**Sulfur Compounds**
4-Methoxy-2-methyl-2-butanethiol	-3.5	-3.9	**Sulfur Compounds**
3-Methyl-2-butenethiol	-3.5	-3.8	**Sulfur Compounds**
Squalene	-7.9	-8.5	**Terpenic Hydrocarbons**
Eremophilone	-6.9	-8.3	**Terpenic Hydrocarbons**
6,10-Dimethyl-l-undecane	-5.9	-6.0	**Terpenic Hydrocarbons**
γ-tocopherol	-8.1	-9.6	**Tocopherols**
α-tocopherol	-8.2	-9.4	**Tocopherols**
β-tocopherol	-8.7	-9.2	**Tocopherols**
δ-tocopherol	-8.5	-8.7	**Tocopherols**
D-(+)-raffinose	-7.2	-7.5	**Triasaccharides**
Maltotriose	-7.0	-7.3	**Triasaccharides**
L-rhamnose	-5.5	-5.5	**Triasaccharides**
β-amyrin	-5.6	-11.0	**Triterpene Alcohols**
28-hydroxytaraxerol	-4.3	-10.7	**Triterpene Alcohols**
Linalool	-0.9	-10.7	**Triterpene Alcohols**
Germanicol	-8.6	-10.5	**Triterpene Alcohols**
24-methylene-24-dihydrolanosterol	-7.1	-10.3	**Triterpene Alcohols**
(24Z)-24-ethylidene-dihydrolanosterol	-7.0	-10.3	**Triterpene Alcohols**
Cyclosadol	-6.4	-10.3	**Triterpene Alcohols**
Lupeol	-3.5	-10.3	**Triterpene Alcohols**
Dammaradienol	-6.6	-10.2	**Triterpene Alcohols**
Tirucallol	-6.3	-10.1	**Triterpene Alcohols**
24-methylene-cycloartenol	-7.0	-10.0	**Triterpene Alcohols**
7, 24-tirucalladienol	-7.4	-9.9	**Triterpene Alcohols**
Cyclobranol	-6.8	-9.9	**Triterpene Alcohols**

28-nor-β-amyrin	-5.2	-9.9	**Triterpene Alcohols**
4,4-dimethyl-5α-stigmasta-7,24Z(241)-dien-3β-ol	-7.1	-9.8	**Triterpene Alcohols**
4α,14α-Dimethylstigmasta-8,24(24)-dien-3β-ol	-8.1	-9.7	**Triterpene Alcohols**
24-methylene-24-dihydroparkeol	-7.6	-9.7	**Triterpene Alcohols**
Bacchar-12,21-dien-3β-ol	-6.6	-9.7	**Triterpene Alcohols**
4,4-dimethyl-5α-stigmast-7-en-3β-ol	-6.5	-9.7	**Triterpene Alcohols**
Butyrospermol	-5.8	-9.5	**Triterpene Alcohols**
α-amyrin	-5.6	-9.5	**Triterpene Alcohols**
β-amyrone	-7.8	-9.4	**Triterpene Alcohols**
Taraxasterol	-6.9	-9.4	**Triterpene Alcohols**
Cycloartenol	-8.0	-9.2	**Triterpene Alcohols**
Agrostophyllinol	-5.0	-9.2	**Triterpene Alcohols**
Methyl 2α,3β-diacetoxyolean-12-en-28-oate	-6.8	-9.1	**Triterpene Alcohols**
Taraxerol	-5.2	-9.1	**Triterpene Alcohols**
3-epi-betulin	-4.5	-9.1	**Triterpene Alcohols**
Ψ-taraxasterol	-4.4	-9.1	**Triterpene Alcohols**
24-methylene-24-dihydroparkenol	-6.2	-9.0	**Triterpene Alcohols**
28-nor-α-amyrin	-6.0	-8.9	**Triterpene Alcohols**
3-epi-lupeol	-5.9	-8.8	**Triterpene Alcohols**
Parkeol	-2.5	-8.8	**Triterpene Alcohols**
δ-amyrin	-4.4	-8.0	**Triterpene Alcohols**
Methyl 3β-acetoxyolean-12-en-28-oate	-0.1	-7.1	**Triterpene Alcohols**
Lupenone	-5.2	-5.9	**Triterpene Alcohols**

Corosolic acid	-4.3	-10.3	**Triterpenic Acids**
Urs-2β,3β-dihydroxy-12-en-28-oic acid	-3.9	-10.2	**Triterpenic Acids**
Ursolic acid	-3.1	-9.7	**Triterpenic Acids**
Maslinic acid	-5.1	-9.5	**Triterpenic Acids**
Oleanolic acid demethyl	-4.5	-9.5	**Triterpenic Acids**
3-epi-betulinic acid	-5.5	-8.9	**Triterpenic Acids**
Betulinic acid	-5.5	-8.9	**Triterpenic Acids**
Pomolic acid	-4.0	-8.9	**Triterpenic Acids**
Oleanolic acid	-3.4	-8.8	**Triterpenic Acids**
Erythrodiol	-3.6	-8.6	**Triterpenic Dialcohols**
Uvaol	-3.2	-8.6	**Triterpenic Dialcohols**
Geranylgeranyl oleate C18:1	-8.2	-8.1	**Wax Esters**
Geranylgeranyl C20:1	-8.1	-8.0	**Wax Esters**
Geranylgeranyl C24:0	-8.1	-7.8	**Wax Esters**
Phytyl oleate C18:1	-7.8	-7.8	**Wax Esters**
Geranylgeranyl oleate C18:0	-7.6	-7.7	**Wax Esters**
Geranylgeranyl C20:0	-8.1	-7.6	**Wax Esters**
Geranylgeranyl C22:0	-7.9	-7.6	**Wax Esters**
Phytyl C20:1	-7.6	-7.6	**Wax Esters**
Phytyl C20:0	-7.6	-7.5	**Wax Esters**
Phytyl C24:0	-7.5	-7.4	**Wax Esters**
Phytyl C22:0	-7.5	-7.3	**Wax Esters**
Wax ester 44:1 (16:0-28:0)	-7.4	-7.3	**Wax Esters**
Phytyl oleate C18:0	-7.6	-7.1	**Wax Esters**
Wax ester 44:1 (18:1-26:0)	-7.4	-7.1	**Wax Esters**
Wax ester 42:1 (16:1-26:0)	-7.3	-7.1	**Wax Esters**
Wax ester 46:1 (18:1-28:0)	-7.3	-7.1	**Wax Esters**
Wax ester 40:1 (18:1-22:0)	-7.1	-7.1	**Wax Esters**
Wax ester 46:0 (14:0-32:0)	-6.9	-7.1	**Wax Esters**
Wax ester 44:0 (16:0-28:0)	-7.3	-7.0	**Wax Esters**
Wax ester 42:0 (16:0-26:0)	-7.2	-7.0	**Wax Esters**
Wax ester 44:1 (20:1-24:0)	-7.2	-7.0	**Wax Esters**
Wax ester 38:0 (18:0-20:0)	-7.0	-7.0	**Wax Esters**
Wax ester 40:0 (20:0-20:0)	-7.0	-7.0	**Wax Esters**

Wax ester 42:0 (24:0-18:0)	-7.4	-6.9	**Wax Esters**
Wax ester 38:0 (20:0-18:0)	-7.3	-6.9	**Wax Esters**
Wax ester 42:1 (18:1-24:0)	-7.3	-6.9	**Wax Esters**
Wax ester 46:0 (20:0-26:0)	-7.2	-6.9	**Wax Esters**
Wax ester 40:0 (18:0-22:0)	-7.1	-6.9	**Wax Esters**
Wax ester 42:0 (18:0-24:0)	-7.1	-6.9	**Wax Esters**
Wax ester 46:0 (18:0-28:0)	-7.0	-6.9	**Wax Esters**
Wax ester 40:0 (14:0-26:0)	-7.3	-6.8	**Wax Esters**
Wax ester 40:0 (16:0-24:0)	-7.3	-6.8	**Wax Esters**
Wax ester 46:0 (22:0-24:0)	-7.2	-6.8	**Wax Esters**
Wax ester 40:1 (16:1-24:0)	-7.0	-6.8	**Wax Esters**
Wax ester 44:0 (22:0-22:0)	-6.9	-6.8	**Wax Esters**
Wax ester 46:0 (24:0-22:0)	-6.9	-6.8	**Wax Esters**
Wax ester 44:0 (18:0-26:0)	-6.7	-6.8	**Wax Esters**
Wax ester 42:0 (20:0-22:0)	-7.3	-6.7	**Wax Esters**
Wax ester 38:0 (12:0-26:0)	-7.0	-6.7	**Wax Esters**
Wax ester 42:0 (14:0-28:0)	-7.4	-6.6	**Wax Esters**
Wax ester 38:0 (14:0-24:0)	-7.3	-6.6	**Wax Esters**
Wax ester 46:0 (16:0-30:0)	-7.3	-6.6	**Wax Esters**
Wax ester 38:0 (16:0-22:0)	-7.2	-6.6	**Wax Esters**
Ethyl oleate	-6.5	-6.6	**Wax Esters**
Methyl oleate	-6.6	-6.5	**Wax Esters**
Methyl stearate	-6.7	-6.4	**Wax Esters**
Methyl palmitate	-6.5	-6.2	**Wax Esters**
Neoxanthin	9.0	-3.0	**Xanthophylls**
Mutatoxanthin	25.0	3.0	**Xanthophylls**
Luteoxanthin	30.9	6.7	**Xanthophylls**
β-cryptoxanthin	20.1	7.8	**Xanthophylls**
Antheraxanthin	28.2	8.0	**Xanthophylls**
Violaxanthin	27.5	9.1	**Xanthophylls**
